# An overview of chemo- and site-selectivity aspects in the chemical conjugation of proteins

**DOI:** 10.1098/rsos.211563

**Published:** 2022-01-26

**Authors:** Charlotte Sornay, Valentine Vaur, Alain Wagner, Guilhem Chaubet

**Affiliations:** Bio-Functional Chemistry (UMR 7199), LabEx Medalis, University of Strasbourg, 74 Route du Rhin, Illkirch-Graffenstaden 67400, France

**Keywords:** bioconjugation, proteins, chemoselectivity, site-selectivity, synthetic chemistry

## Abstract

The bioconjugation of proteins—that is, the creation of a covalent link between a protein and any other molecule—has been studied for decades, partly because of the numerous applications of protein conjugates, but also due to the technical challenge it represents. Indeed, proteins possess inner physico-chemical properties—they are sensitive and polynucleophilic macromolecules—that make them complex substrates in conjugation reactions. This complexity arises from the mild conditions imposed by their sensitivity but also from selectivity issues, *viz* the precise control of the conjugation site on the protein. After decades of research, strategies and reagents have been developed to address two aspects of this selectivity: chemoselectivity—harnessing the reacting chemical functionality—and site-selectivity—controlling the reacting amino acid residue—most notably thanks to the participation of synthetic chemistry in this effort. This review offers an overview of these chemical bioconjugation strategies, insisting on those employing native proteins as substrates, and shows that the field is active and exciting, especially for synthetic chemists seeking new challenges.

## 1. Introduction

**Figure 1.**
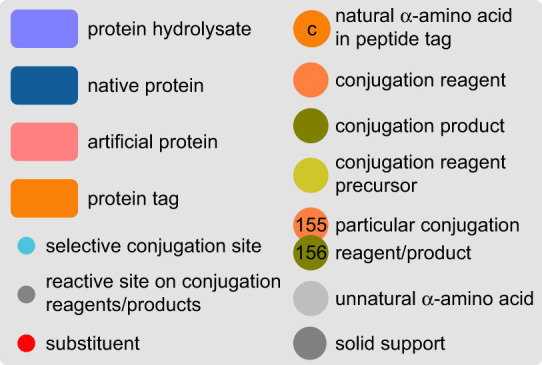
Legend of all graphical elements used in the manuscript.

Bioconjugation can be defined as the covalent coupling between a biomolecule—in this case, a protein—and any other molecule, including another biomolecule. The resulting bioconjugates can display either the combined properties of its separate constituents—as in the case of fluorophore-conjugated proteins for example—or a new set of properties that cannot be explained by simply considering the two conjugated entities separately—as is the case with toxins leading to non-toxic but still immunogenic toxoids upon chemical conjugation. Because of this modularity, applications of protein conjugation are plentiful, and too vast to be cited exhaustively. One can, however, mention the therapeutic field, which has become the object of intense scrutiny over the past decades, with the emergence of immunoconjugates—that is, bioconjugates of immunoglobulins, and more specifically antibodies—for the treatment of several types of cancers. This increased attention toward protein conjugates went hand-in-hand with improved understanding of proteins reactivity and more advanced bioconjugation tools, currently dominated by enzymatic and chemical strategies, the latter being the central topic of this review. Because of proteins' polynucleophilic nature—with the possible exception of asparagine and glutamine, all reactive groups found on the side-chains of proteinogenic amino acids are nucleophiles to some extent, large in size compared to classical small molecules and sensitivity—limiting the use of organic solvents; narrowing the range of applicable temperatures; forcing us to ban strong oxidizing/reducing agents, bases and acids, etc.—their chemical conjugation represents a challenge on many aspects. Similar to the French literary movement Oulipo using constrained writing techniques to foster inventiveness and innovation, the rigid framework imposed by proteins' physico-chemical properties has been seen by many synthetic chemists as an exciting playground for the development of new chemical tools. In this regard, chemoselectivity—targeting selectively only one family of amino acid residues; and site-selectivity—targeting a single copy of a precise amino acid—represent the central issues to be addressed, in order to develop techniques that would be reproducible and lead to homogeneous, well-defined protein conjugates. After decades of work and investigation, myriad solutions to these two challenges have been offered, which will be summarized in this review. A large part of this document will be devoted to site-selective strategies—sometimes also referred to as ‘site-specific’, see §4 for a semantic discussion—and, more specifically, to that employing native proteins, as we believe this is where the pinnacle of chemical innovation is. In this particular section, we limited ourselves to publications in which site-selectivity was clearly demonstrated, evidenced and supported by analytical data—essentially peptide mapping studies by LC/MS–MS. While this review does not intend to be exhaustive, we tried to cover the field as extensively as possible in order to give the reader a good overview of the century spanning chemical bioconjugation, from its early development in the tanning industry to the most recent site-selective photoredox-catalysed techniques, thus providing a broader picture than the most recent reviews on a similar topic [1–5]. A legend explaining the various graphical elements used in this manuscript is provided in figure 1 to facilitate the reader's understanding.

## 2. Chemoselective strategies

### 2.1. Early developments

Initially based on empirical observations without a clear understanding of the processes taking place at the molecular level, the chemical modification of proteins had long been present and used for practical purposes before becoming a topic of interest in chemical biology. In the mid-nineteenth century, chromium(III) salts were thus employed in the tanning industry to produce leathers by cross-linking collagen proteins in animals' hides [6]. In the early 1920s, formaldehyde was used for the modification of toxins related to a certain number of bacterial diseases. In this chemical process, the bacterial proteins would lose their toxicity while retaining their ability to elicit an immune response, turning them into effective toxoid vaccines [7].

**Figure 2.**
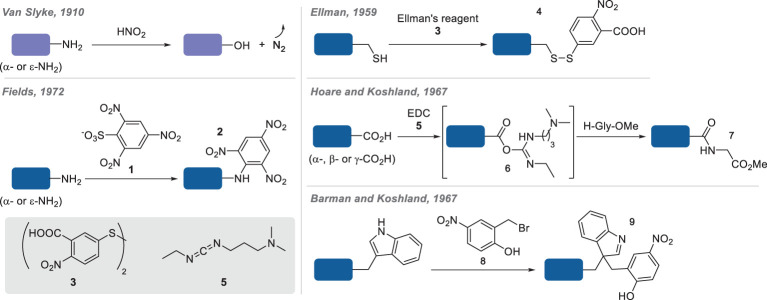
Early developments of chemoselective reagents for the conjugation of proteins.

Over the twentieth century, the improvement of analytical techniques for the characterization of proteins (e.g. cation-exchanger amino acid analyser, ion exchange and gel exclusion chromatography) coupled with the definitive mapping of all proteinogenic amino acids (identification of methionine in 1922; threonine in 1925) have highly contributed to the development of new techniques for the chemical modification of proteins [6]. Several functional group-selective—i.e. *chemoselective*, *vide infra*—methods were thus described between the late 1950s and early 1970s, helping with the determination of proteins' primary structures (figure 2). Variations of the original Van Slyke's procedure, consisting of measuring the development of gaseous dinitrogen from the reaction of primary amines in proteins hydrolysates with nitrous acid, were developed for the quantification of amine groups in intact proteins [8,9]. For instance, Fields reported the use of trinitrobenzenesulfonate **1** (TNBS) for the formation of trinitroanilines **2** that could be quantified by measuring their absorbance at 420 nm [10].

Similar to sodium nitroprusside that gives a red colour in the presence of thiols in protein, Ellman's reagent (5,5′-dithiobis-(2-nitrobenzoic acid), DTNB; **3**) was developed in 1959 to quantify the concentration of thiols in various biological samples by measuring the absorbance of the highly chromogenic 5-thio-2-nitrobenzoic acid (TNB) **4** at 412 nm [11]. In 1967, Hoare & Koshland [12] proposed to modify all carboxylate groups (i.e. aspartate and glutamate side-chains as well as *C*-terminus) in insulin, lysozyme and ribonuclease with the water-soluble 1-ethyl-3-(3-dimethylaminopropyl)carbodiimide (EDC; **5**). The resulting *O*-acylisoureas **6** were then reacted with the nucleophilic glycine methyl ester, leading to coupling products **7**. The protein was then hydrolysed and the extra-glycine content—as the direct reflection of carboxylate content—determined by an amino acid analyser. For the determination of tryptophan residues in proteins, Barman & Koshland [13] proposed to label those residues selectively with 2-hydroxyl-5-nitrobenzyl bromide **8**, forming coloured complexes **9** whose absorbance at 410 nm could be directly correlated to the number of indoles present in the protein.

The emergence of these chemoselective bioconjugation reagents also went hand-in-hand with a better understanding of proteins' functions. Following the idea that the residue(s) responsible for the activity of enzymes might be identified on the basis of their reactivity towards specific electrophiles [14,15], Balls & Jansen [16] showed in 1952 that diisopropyl fluorophosphate could inhibit several proteases such as α-chymotrypsin, trypsin or cholinesterase by phosphorylating selectively the serine residue present in their active site.

All these innovations facilitated the characterization of modified proteins and led to a better understanding of the reagents’ chemoselectivity. In this context, chemoselectivity can be seen as the selective modification of a side-chain functional group borne by the amino acids constitutive of the protein—e.g. lysines' ε-amines—in the presence of others with similar reactivity—i.e. *N*-terminal α-amines, cysteines’ thiols and nucleophiles in general.

However, early strategies tended to be hampered by limited conversion [6]. While employing more vigorous conditions (longer reaction time, larger excess of reagent, higher reaction temperature, etc.) could help overcome this limitation, it also came with the risk of eroding the chemoselectivity and affecting the conformation and activity of the protein. Consequently, this urged scientists to develop new families of reagents that could be employed under milder conditions—aqueous media, narrower range of temperature and pH, etc. —to preserve the integrity of the biomolecule. The following section will thus present briefly the most classical chemoselective strategies that have been developed over the past decades.

### 2.2. Modern methods for the chemoselective conjugation of proteins

Alongside the improvement of analytical techniques for the characterization of proteins, the development of mild strategies and new reagents for their chemoselective modification arose. Those methods relied heavily and essentially on the modification of the two most nucleophilic amino acids found in proteins: lysine and cysteine.

#### 2.2.1. Lysine residues

Lysine is one of the most abundant amino acids in proteins [17]. Composed of a linear four-carbon chain terminated with a primary amino group, lysine, along with arginine and histidine residues, contributes to the overall net positive charge of proteins. In comparison with the *N*-terminus α-amino group, the ɛ-amine of lysine possesses a slightly higher ionization point (p*K*_a_ of ɛ-amine is comprised between 9.3 and 9.5, α-amine between 7.6 and 8.0), thus favouring the formation of positively charged ammonium groups at physiological pH. Due to this dominant ionic character, lysines' side-chains are thus generally exposed to solvent, at the surface of proteins, making them easily accessible for chemical derivatization. Several chemoselective methods were hence described, generally conducted at alkaline pH between 8 and 10 in a wide range of buffers—including Tris [18], counterintuitively—and revolving mainly around three families of reagents: carbonyls, activated esters or iso(thio)cyanate [19–23].

As one of the fundamental reactions in organic chemistry, primary amines condense reversibly with carbonyls to form imines. Being disfavoured in aqueous medium and leading to unstable adducts, imine formation is rarely, if ever, used as a standalone bioconjugation technique but rather as a way to generate reactive intermediates that will participate in a subsequent irreversible step. Reductive amination is an example of such a two-step transformation, sodium cyanoborohydride acting generally as the reducing agent in the irreversible step leading to secondary amines [19,24], but many more bioconjugation reactions employing imines as intermediates have been developed, which will be discussed later in this review.

**Figure 3.**
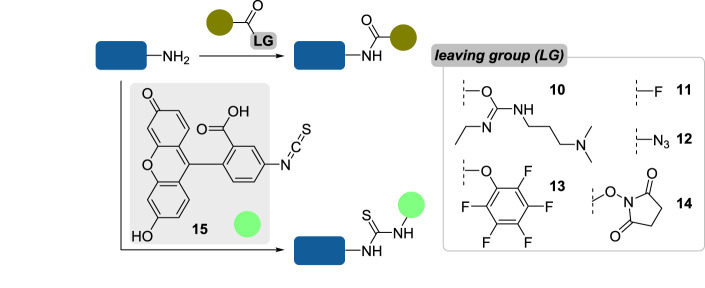
Classical bioconjugation methods for the chemoselective labelling of lysine residues.

As an alternative to carbonyls, primary amines can also react with activated carboxylates to yield amides, the most-employed strategy in the field of protein bioconjugation (figure 3). Of the myriad reagents reported, one can cite *O-*acylisoureas—such as **10**, transiently generated from EDC **5**—acyl halides and azides (**11** and **12**, respectively), pentafluorophenyl esters **13** or *N*-hydrosuccinimidyl (NHS) esters **14** [25–30]. The latter are by far the number one reagents for lysine conjugation, thanks to their facile preparation and mild reaction conditions associated with their use—neutral to near-neutral pH, room temperature, various buffers tolerated, reaction times usually comprised between dozens of minutes and few hours—leading to exceedingly stable conjugates (the half-life of amide bonds in neutral aqueous solution has been estimated up to 600 years) [31] albeit suppressing a permanent charge at the surface of the protein and thus altering its isoelectric point. To address this limitation, imidoesters were developed, yielding positively charged amidine groups at neutral pH [32,33]. However, the amidine bond was shown to be unstable at elevated pH, thus limiting the use of imidoesters as conjugation reagents [34].

Even though NHS esters are seen as the gold standard of lysine conjugation reagents, an inevitable drawback of employing reactive electrophiles in aqueous media is their competitive hydrolytic decomposition, which affects the efficiency of the conjugation step. Increasing the pH of the reaction to work under more alkaline conditions has been proposed to facilitate amine acylation, but this was also accompanied with accelerated hydrolysis of NHS esters, with a typical half-life of just 10 min at pH 8.6 and 4°C [34]. While similar issues can be observed with isocyanates, making them disregarded reagents in bioconjugation, isothiocyanates are much more resistant towards hydrolysis and have been classically employed for the chemical transformation of amines to thioureas. The most famous member of this family of reagents is probably fluorescein isothiocyanate (FITC, **15**), frequently used for the fluorescent labelling of proteins [35–37]. Even though the rate of hydrolysis of isothiocyanates was found to be slower than that of NHS esters, thioureas tend to be less stable than amide bonds, explaining why activated esters—and reagents or strategies leading to amide conjugation product in general—remain unrivalled for the chemoselective modification of amines in proteins.

#### 2.2.2. Cysteine residues

**Figure 4.**
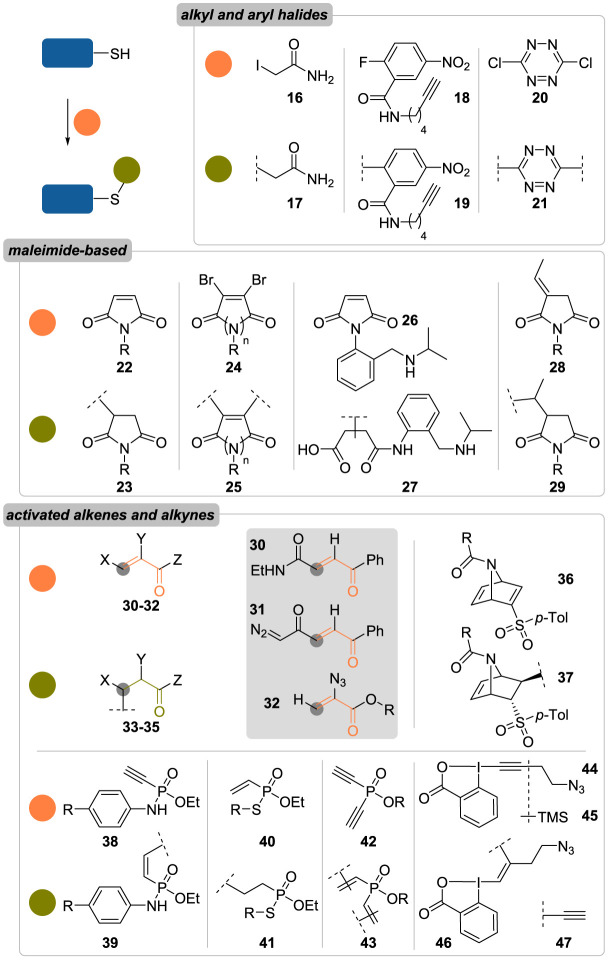
Overview of classical methods for the chemoselective conjugation of cysteine residues (R = alkyl chain; *p*-Tol = *para*-toluyl).

Because of their low abundance (1.5%) and the high reactivity of their sulfhydryl group, cysteine residues are one the most convenient targets in bioconjugation [34,38]. The ionization of cysteines’ side-chain occurs at alkaline pH (thiol's p*K*_a_ 8.8–9.1) generating a negatively charged and more nucleophilic thiolate residue. Alkyl halides, disulfides and maleimides—and more generally electron-deficient alkenes and alkynes—are the three main classes of reagents used for the chemoselective conjugation of cysteines (figure 4) [20–22,39].

Alkyl halides and especially haloacetamides have been employed for the labelling of thiols in peptides and proteins for decades. Alongside 4-vinylpyridine and acrylamide, these reagents have traditionally been used to alkylate cysteines prior to Edman degradation and automated amino acid analysis [40,41], and are still a valuable tool for mass spectrometry analyses, where a preliminary conjugation step with iodoacetamide **16** is routinely performed before peptide mapping studies [42]. While alkyl halides are prone to side reactions with other nucleophilic amino acids (e.g. histidine, methionine, lysine and *N*-terminal residues) [43], the selectivity and reactivity of those reagents can be tuned by either varying the halide—order of reactivity: I > Br > Cl > F—or playing with reaction conditions. Cysteine selectivity has thus been demonstrated for iodoacetate derivatives when used as limiting reagents under slightly alkaline conditions (pH 8.2) for a few minutes. In the same vein, aryl halides were also described for the modification of thiols through aromatic nucleophilic substitution, generating aryl thioether species. Successful thiol conjugation when employing such reagents relies heavily on stereoelectronic effects favouring the rate-determining *ipso*-addition step; as such, aryl fluorides and chlorides bearing strongly electron-withdrawing groups in *ortho*/*para* positions—such as **18** and **20**—form the overwhelming majority of substrates reported to date [44].

As discussed previously, Ellman's reagent **3** (figure 2) and activated disulfide reagents in general have also proven to be highly cysteine-selective, reacting with this residue via disulfide exchange and yielding hydrolytically stable conjugates. However, their inherent reactivity makes them prone to reduction and disulfide exchange in thiol-rich, reductive environments, where their half-lives typically never exceed 1 h [45].

Of all cysteine-selective reagents reported to date, the most studied and most employed remains maleimide **22** (figure 4) [46]. Reacting with thiolates via a thio-Michael addition to form thioethers **23** [46,47], maleimides display best chemoselectivity at pH values comprised between 6.5 and 7.5 (competitive aza-Michael addition starts to occur at pH greater than 8.5) [48–50] and fast kinetics, with second-order rate constants oscillating between 100 and 1000 M^−1^ s^−1^, depending on the structure of the thiol and the pH [46,51,52]. While extremely effective, maleimide conjugation is still accompanied by certain limitations: despite a good stability, maleimide adducts can undergo retro-Michael addition and thiol exchange under physiological conditions [45]; maleimides reagents can be prone to hydrolysis and yield ring-opened maleamic acids that are unreactive towards thiol addition; and maleimides are not amenable to multiple functionalization, due to their limited number of reactive sites.

With the intent of improving the latter point, Caddick and co-workers [53–55] reported in the early 2010s new families of structurally related reagents, based on maleimide (**24**; *n* = 1) and pyridazinedione (**24**, *n* = 2) skeletons, in which the incorporation of two nucleofuges, i.e. halides or thiophenolates, across the reactive C=C bond allowed two successive addition–elimination sequences, resulting in doubly functionalized conjugates **25**.

However, these were still found to suffer from *retro*-thio-Michael addition as thiol exchange was demonstrated in the presence of an excess of glutathione or 2-mercaptoethanol.

To minimize the reversibility of the *retro*-thio-Michael addition, several studies have tried to facilitate and accelerate ring hydrolysis after thiol addition, as the resulting thiomaleamic acids proved to be resistant to thiol exchange [45,56]. One notable example are *o*-CH_2_NH^i^Pr phenyl maleimides **26**, reported by Kalia *et al.* [57], whose corresponding thiol conjugates were rapidly hydrolysed (half-lives typically less than 1 h) to ring-opened derivatives **27** thanks to both the electron-withdrawing effect of the phenyl ring and the intramolecular maleimide activation from the appended ammonium group. In a previous study, they had also demonstrated that exocyclic olefinic pseudo-maleimides **28** led to more stable conjugates over time than classical maleimides, with negligible degrees of thiol exchange being detected on a bovine serum albumin-conjugate after 7 days of incubation at 37 °C in the presence of a 40-fold excess in *N*-acetyl cysteine [58].

Maleimides have thus been the object of intense scrutiny and can safely be considered as the mainstay of cysteine conjugation. But despite this dominant position, research towards the development of other thiol-selective reagents based on electron-deficient alkenes (and alkynes) has been quite active—and continues to be [59].

Acrylic derivatives **30** have thus been identified as effective Michael acceptors for the modification of cysteines residues (figure 4). These reagents demonstrated excellent chemoselectivity and fast kinetics for the conjugation of various proteins—including an engineered antibody; trastuzumab LC-V205C [60]—and paved the way towards the development of analogous compounds incorporating diazo or azido handles (**31** and **32**, respectively) allowing further functionalization of the protein conjugates [61,62].

Vinyl sulfones have also been known for decades as effective Michael acceptors for the modification of proteins, leading to exceedingly stable adducts (the adduct resulting from the reaction between cysteine and ethyl vinyl sulfone showed less than 15% hydrolysis after 24 h at 110 °C in 6 N HCl!) [63] despite poor chemoselectivity often being observed, with lysine and histidine residues competing with cysteines for alkylation, especially under alkaline pH (i.e. greater than or equal to 8.0) [63–65]. Interestingly, by embedding the vinyl sulfone motif into an azabicyclic system, as in reagent **36**, excellent cysteine selectivity was attained by the Bernardes group for the rapid conjugation of few mutant proteins with single solvent-exposed cysteine residue [66]. However, the resulting adducts **37** suffered from *retro*-Diels–Alder reaction, leading to payload fragmentation over prolonged incubation at 37 °C in plasma.

In a similar vein, Hackenberger and co-workers [67,68] reported the use of ethynylphosphonamidates **38** and vinylphosphonothiolates **40** for either azide-to-thiol or thiol-to-thiol conjugation, respectively. Even though kinetics were rather slow compared to maleimide (less than or equal to 0.6 M^−1^ s^−1^), both reagents showed excellent chemoselectivity at pH 8.5 and led to various protein conjugates, including protein hetero-dimers, all of which were stable for more than 4 days in different media (i.e. PBS, cell lysates, serum and highly reductive environment). Elaborating on this P(V) platform, the same group recently reported phosphinates **42**, incorporating two terminal alkynes and thus offering protein cross-linking possibilities [69]. Applied to the disulfide rebridging of trastuzumab, half-antibody was observed as the main conjugation product; however, a side-reaction invariably competing with the expected inter-chain conjugation in these strategies (see §3.2.2. for further details).

Finally, a more recent application of activated alkynes for the rapid and chemoselective conjugation of cysteine residues in a myriad of peptides and proteins was reported by Waser and co-workers, who described two structurally related ethynylbenziodoxolones (EBX) reagents. Azide-bearing EBX reagent **44** was used for the introduction of azides into proteins in the form of conjugates **46** that could be sequentially modified by SPAAC and Suzuki–Miyaura cross-coupling reactions [70,71]. Interestingly, a different reactivity was observed with trimethylsilyl derivative **45**: following a preliminary *in situ* desilylation, thiol-yne β-addition was followed by an α-elimination of the iodide moiety leading to a transient carbene that underwent a 1,2-sulfur shift, resulting in the generation of a terminal thioalkyne **47** that could be further modified by CuAAC [72].

This quick overview of the two main chemoselective strategies shows how the field of protein bioconjugation has flourished in the recent years, offering now dozens—if not hundreds—of diverse ways to label selectively amines and thiols. In response to this booming expansion, the scientific community took an increasing interest in the domain, which fostered creativity and promoted the development of alternative methods for the conjugation of less nucleophilic amino acid residues.

#### 2.2.3. Other residues

Tyrosine emerged as an attractive target because of its limited abundance at the protein surface and its electron-rich phenol(ate) side-chain. Even though original strategies have recently appeared [73], tyrosine conjugation has essentially relied on nucleophilic aromatic *ortho*-substitution since its early days in the 1900s and Hermann Pauly's work on the use of diazobenzenesulfonic acid—which would later be remembered as Pauly's reagent—for the colorimetric determination of tyrosine (and histidine) residues in proteins [74]. While far from being optimal, this study laid the groundwork toward the development of more chemoselective diazonium reagents, a quest that is still being pursued nowadays, more than a hundred years after their first report [75]. Diazonium reagents only represent the tip of the tyrosine-conjugation iceberg, as dozens of other strategies and reagents have been developed, all summarized in two recent, comprehensive and independent reviews by Chudasama and co-workers [76] and Gouin and co-workers [77]. Another alternative target for chemoselective conjugation was brought to light by the study of advanced glycation end-products, which showed that glyoxal—and 1,2-dicarbonyls in general—were selectively reacting with arginines' guanidinium groups [78–80], a strategy recently used by our team for the production of immunoconjugates [81].

Pursuing this continuous effort toward mastering the chemoselective conjugation of protein, several strategies have recently focused on the functionalization of less nucleophilic—and thus more challenging—residues, such as methionine or serine. However, because these residues tend to be less abundant at the surface of proteins than those previously discussed, the border between chemoselectivity and site-selectivity becomes more porous and less pertinent. For this reason, several chemoselective strategies will be discussed in the section dedicated to site-selective conjugation (see §4).

Altogether, the development of all these reagents and methods led to the generation of countless protein conjugates and myriad applications: the incorporation of fluorophores helped with the understanding of cellular mechanisms by analysing the trafficking of labelled proteins [82] polyethylene glycol (PEG) chains functionalization, also known as PEGylation, yielded less-immunogenic and more plasma-stable proteins [83,84]; conjugation of cytotoxic drugs to a carrier protein led to diminished systemic toxicity and facilitated their targeted delivery to malignant cells [85,86].

In the latter case, and in particular for antibody-drug conjugates (ADCs), the conjugation method was found to be one of the determinant parameters of the efficacy of the resulting conjugates [86,87]. While chemoselective strategies led to highly potent therapeutics—notably the marketed ADCs Mylotarg, Kadcyla and Besponsa, whose cytotoxic payloads were incorporated via lysine conjugation—it also generated complex and highly heterogeneous samples. Composed of regioisomeric mixtures of thousands of species with various degrees of conjugation, all of which possess different pharmacokinetics, chemoselective strategies are not the best ally in insuring batch-to-batch reproducibility and consistency of immunoconjugates production. This is a direct consequence of the large size of antibodies (approx. 150 kDa) and therefore of the elevated number of potential conjugation sites. Gautier *et al.* [88] best epitomized this issue in a seminal work in 2015, where they demonstrated that 69 of the 88 possible reactive amine groups (84 lysines and 4*N*-termini) on immunoglobulins G (IgG) could be conjugated with NHS esters. All things being equal, based on this reactivity profile this means that an IgG conjugated with six payloads would actually correspond to a mixture of approximately 120 million regioisomers.

Therefore, the next logical step for researchers who wanted to solve the heterogeneity issues of chemoselective protein bioconjugation was to develop *regioselective*—i.e. *site-selective*—methods and reagents; that is, methods and reagents that would lead to the conjugation of one precise residue among its many copies. This was highly desired for therapeutic applications, for which homogeneous conjugates could widen the therapeutic window and potentially improve the efficacy of the treatment [86,87,89]. Because site-selectivity can be seen as the next logical step after chemoselectivity in the ranking of complexity in selectivity—which could roughly be summarized as chemoselectivity < site-selectivity < protein selectivity—the knowledge gained during the study of protein reactivity in the former field helped develop the latter. Thus, and despite isolated examples showing the opposite [90], it is not surprising to note that the vast majority of site-selective strategies employ chemoselective reagents. These can be structurally tuned and improved for the purpose of single-site conjugation, or used as they are but under particular conditions (e.g. lowered temperatures, acidic conditions to favour *N*-terminus conjugation) or on proteins for which the distinction between chemoselectivity and site-selectivity is no longer relevant (i.e. proteins with a single solvent-accessible cysteine residue). Looking closer at the types of proteins employed in site-selective conjugation studies, two main families can be distinguished: artificial proteins—in which specific amino acids or sequences that are naturally absent from the parent protein have been introduced—or native proteins, whose primary sequences have not been edited.

## 3. Site-selective strategies

Site-selectivity and regioselectivity can be considered as synonyms and defined as the preferential conjugation of a single residue on the protein of interest (POI). While site-specificity is also often used in the literature to describe such a process [91–93], selectivity and specificity possess distinct definitions in the context of organic chemistry that should make them not interchangeable [94]. However, for the sake of clarity and as the majority of strategies discussed hereafter can be considered site-selective, we will *only* use this term whenever describing a reaction favouring one or a limited number of conjugation sites on the POI, irrespective of the structure of the reagent and of the reaction mechanism.

Currently, site-selective strategies are dominated by artificial proteins, in which an unnatural amino acid (UAA)—which should be understood in this case as a residue not naturally present in the parent protein—residue or peptide sequence has been incorporated via genetic engineering, and whose reactivity is orthogonal to that of natural residues. Using tailored reactions, the selective labelling of these exogenous entities is greatly favoured and yields homogenous protein conjugates.

### 3.1. Site-selective labelling of artificial proteins

As previously discussed, three main strategies have been investigated for the site-selective conjugation of artificial proteins, depending on the nature of the exogenous group incorporated: a natural amino acid, a non-canonical amino acid and peptide or protein tags.

#### 3.1.1. Incorporating natural α-amino acids

One of the first strategies explored was the introduction on the POI of poorly abundant yet natural amino acids, on positions where further functionalization would be possible without altering the structure and function of the biomolecule, such as *N-* or *C*-terminal positions, or on a loop [95].

Because of its high nucleophilicity and the poor abundance of free thiols in proteins, engineering of (seleno)cysteines by site-directed mutagenesis has been extensively employed [96,97]. Indeed, as cysteines are usually found as disulfide bonds in proteins, incorporation of an unpaired residue allowed the modification of the engineered protein via the numerous previously reported chemoselective methods (*vide supra*), but also triggered the development of new strategies.

**Figure 5.**
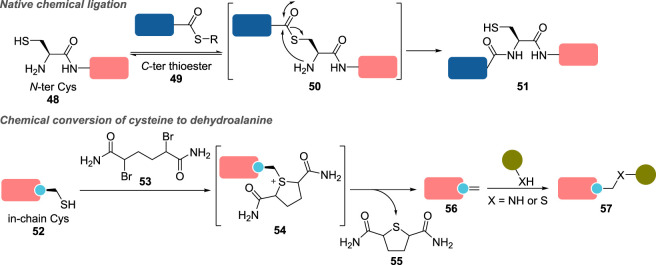
Selected examples of site-selective conjugation strategies using artificial proteins with engineered cysteine residues (R, alkyl or aryl).

*N*-terminal cysteines **48** and their 1,2-amino thiol motif have been selectively modified with thioesters for instance, as best exemplified by native chemical ligation (figure 5) [98,99], but also with tailored reagents bearing a reactive unsaturated carbon centre, such as 2-cyanobenzothiazole and diversely substituted 2-formylphenylboronic acids [95], as we will detail later in §3.2.3 and figure 16.

The development of a chemoselective di-alkylation/elimination cascade also permitted the chemical conversion of in-chain (seleno)cysteines **52** to dehydroalanines (Dha) **56** using 1,4-dihaloalkyl reagents such as **53** [100,101]. The resulting alkene moiety of Dha could then be selectively modified under mild conditions by appropriate nucleophiles, essentially via *aza*- and *thio*-Michael additions, to yield conjugates **57**. This strategy has notably been applied to the generation of chemically defined ADC, using THIOMAB antibodies, a class of artificial proteins developed by Genentech in which key native residues (in this case, the two valine residues V205 of the light chain) have been replaced with cysteines [102].

Besides (seleno)cysteines, other low-abundant amino acids have been engineered on proteins, such as tryptophan, tyrosine, methionine and histidine, but the limited number of chemoselective reactions to conjugate them coupled with competition with naturally occurring residues has limited their widespread utility [96].

Thus, of all natural amino acids, only engineered (seleno)cysteines found valuable applications in site-selective conjugation and the production of homogeneous conjugates. Albeit chemoselective, the conjugation strategies employed to target these residues still had to be finely tuned to limit side reactivity and reach good yields and conversion rates, some critical aspects that can be addressed by employing UAA and bioorthogonal conjugation reactions instead.

#### 3.1.2. Incorporating unnatural α-amino acids

The incorporation of a variety of UAA provided unique chemical handles for the site-selective modification of proteins [95,96,103–105]. Azide, alkyne, alkene, tetrazine and carbonyl-containing amino acids were designed and incorporated into proteins via genetic code expansion [106], offering the possibility to use bespoke reagents to modify these exogenous, unnatural moieties selectively without interfering with any natural functional group; in a *bioorthogonal* manner.

**Figure 6.**
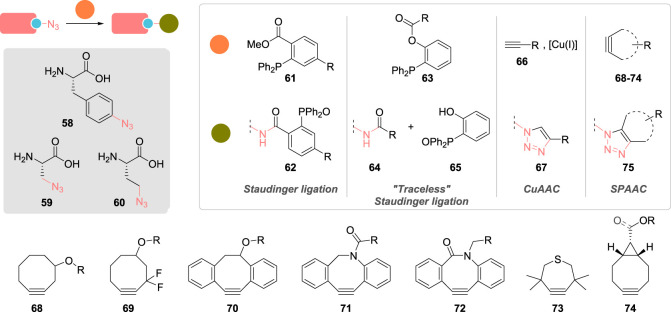
Incorporation of azido amino acids via genetic manipulation in proteins (R, alkyl). The introduced azido group can then be modified by Staudinger ligations, copper-catalysed alkyne-azide cycloaddition (CuAAC) and strain-promoted alkyne-azide cycloaddition (SPAAC).

The Staudinger ligation between an azide and a triarylphosphine reported by Saxon & Bertozzi for the modification of azido-glycoproteins in living cells was the pioneering work demonstrating the vast potential of bioorthogonal reactions for the conjugation of unnatural proteins (figure 6) [107]. This technique and its variations, such as the traceless Staudinger [108], were then extended to the direct modification of mutant proteins that had incorporated *p-*azidophenylalanine **58** [109,110], azidoalanine **59** [111] or azidohomoalanine **60** amino acid residues [111]. However, because of slow kinetics (second-order rate constants classically found in the 10^−3^ M^−1^ s^−1^ range), the retained triarylphosphine oxide appendage in ligation products **62**, the sensitivity of phosphines **61** and **63** toward oxidation and the possibly competing iminophosphorane hydrolysis, the Staudinger ligation progressively lost its interest to the benefit of the copper(I)-catalysed alkyne-azide cycloaddition (CuAAC) and the selective formation of 1,4-triazole conjugates **67** [112–115]. As this rapid reaction (i.e. *k*_obs_ comprised between 10 and 100 M^−1^ s^−1^) was found to be bioorthogonal and highly efficient under physiological conditions, it found myriad applications for the modification of recombinant proteins containing UAA with either azido or alkynyl substituents. Despite the problematic generation of harmful reactive oxygen species by the copper/ascorbate/atmospheric oxygen triad, effective procedures for the CuAAC labelling of live cells have nevertheless been reported [116].

As an alternative to the use of copper catalysts, Bertozzi and co-workers [117] had the revolutionary idea to adapt strain-promoted alkyne-azide cycloaddition (SPAAC) to the field of bioorthogonal chemistry in 2004, showing that cyclooctyne **68** could react rapidly at room temperature with azido-glycoproteins without the need for any catalytic system. Initially reported by Blomquist & Liu [118] before being studied in more detail by Wittig & Krebs [119], this facile reaction between cycloalkynes and azides results from the high reactivity of the bent triple bond, forced out of the ideal 180° bond angle by the cyclic and rigid framework. Because SPAAC kinetics were initially found to be similar to that of the slow Staudinger ligation [112], a number of diversely substituted strained alkynes were developed, playing essentially on stereoelectronic parameters, in order to accelerate the rate of this cycloaddition. Cyclooctynes and cycloheptynes were determined to be the best compromise between stability and reactivity, explaining the prevalence of these skeletons in the various reagents developed over the years; e.g. difluorinated cyclooctyne **69** (DIFO) [120–122], dibenzocyclooctynol **70** (DIBO) [123], dibenzoazacyclooctyne **71** (DIBAC) [124], biarylazacyclooctynone **72** (BARAC) [125], tetramethylthiacycloheptyne **73** (TMTH) [126–128] and bicyclo[6.1.0]nonyne **74** (BCN) [129]. While cycloalkynes are essentially used as small molecular probes to functionalize azide-containing artificial proteins, the reverse approach has also been documented with cyclooctyne-containing UAA being genetically encoded into proteins [130,131].

**Figure 7.**
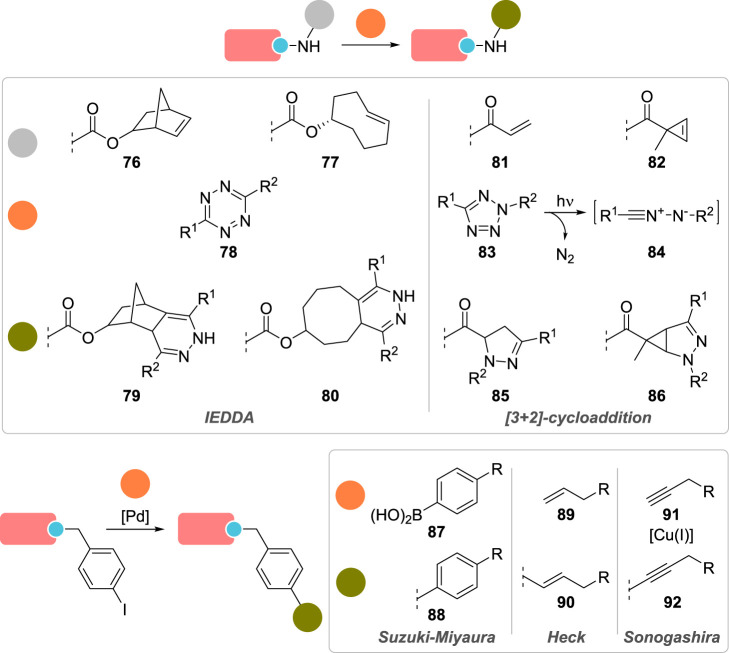
Site-selective modification of UAA on artificial proteins using bioorthogonal reactions. UAA 76, 77, 81 and 82 are lysine derivatives; UAA with *p*-iodobenzyl side-chain are either cysteine or phenylalanine derivatives 121 and 116, incorporated either chemically or by genetic code expansion, respectively.

Parallel to the development of SPAAC for the labelling of proteins, inverse-electron demand Diels–Alder reactions (IEDDA) also emerged as a new promising tool (figure 7). Reacting electron-rich dienes, such as *trans*-cyclooctenes or norbornenes, with tetrazine dienophiles led to exceedingly fast bioorthogonal reactions with second-order rate constants up to 10^5^ M^−1^ s^−1^ [132–134]. New reactive UAA such as **76** and **77** were thus developed and applied to the conjugation of several artificial proteins, including cell-surface, cytosolic and nuclear proteins in live cells [131,135–138].

**Figure 8.**
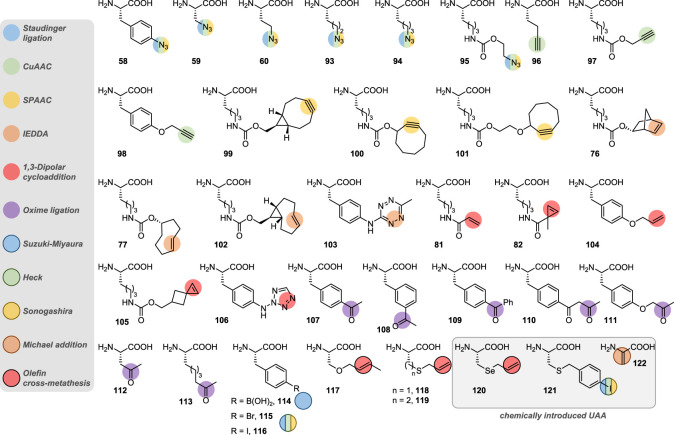
Examples of unnatural amino acids that have been incorporated into artificial proteins alongside the bioorthogonal technique employed to functionalize them.

Another approach to fast bioorthogonal reaction for site-selective protein modification employs the [3 + 2]-cycloaddition between a transient nitrile-imine **84**, generated *in situ* by photo-irradiation of tetrazole **83** and a variety of alkenes, such as **81** and **82** (figure 7) [139]. This spurred the development of various alkenyl and tetrazole UAA (see **104**–**106**; figure 8), which were site-specifically incorporated and labelled in various proteins in live cells [140–143]. While second-order rate constants of this [3 + 2]-cycloaddition were found to be three to four orders of magnitude lower—*viz*. between 10 and 100 M^−1^ s^−1^—than that of IEDDA, it possesses the advantage of a possible spatial and temporal control, as UV irradiation is required to trigger the formation of the reactive nitrile-imine species.

Drifting away from cycloadditions, incorporation of carbonyl groups in recombinant proteins has also been investigated due to their limited occurrence in native proteins and their selective modification with α-nucleophiles, such as alkoxyamines and hydrazines, to form oximes and hydrazones, respectively. Despite not being bioorthogonal *per se*—amines condensation to form imines is also possible—this strategy has still found several applications in chemical biology over the years [144]. While site-selective generation of terminal aldehydes could be easily achieved on proteins possessing *N*-terminal serine or threonine residues, via periodate-mediated oxidative cleavage [145], aldehyde and ketone-containing UAA have also been designed [146,147], such as *p*- and *m*-acetyl-l-phenylalanine **107** and **108**, respectively [148–154], *p*-benzoyl-l-phenylalanine **109** [150,152] and 2-amino-8-oxononanoic acid (KetoK) **113** (figure 8) [155]. However, the slow kinetics associated with carbonyl condensation—second-order rate constants ranging from 10^−4^ to 10^−3^ M^−1^ s^−1^, making this transformation the slowest of all bioorthogonal reactions—coupled with the need for acidic pH to achieve acceptable conversion have limited the use of this labelling technique.

Besides CuAAC, numerous other transition-metal catalysed strategies for the modification of UAA have surfaced in recent years (figure 7) [103,156]. Associated with excellent chemoselectivity, bioorthogonality and high yields, palladium-catalysed cross-couplings between aryl/alkenyl halides and a variety of reactive partners such as boronic acids **87** (Suzuki–Miyaura) [157–165], alkenes **89** (Heck) [166–168] and terminal alkynes **91** (Sonogashira) have been described for site-selective protein conjugation [166,168,169]. Early use of these reactions was hampered by technical difficulties as they needed to proceed in aqueous media and under low protein concentration, necessitating elevated temperatures in certain cases and high catalyst loading that would lead to protein degradation and side reactions, explaining the low yields that often accompanied these cross-couplings [157,158,167,168]. Improvement in this field has essentially been realized thanks to the development of more effective catalysts. The Davis group reported a phosphine-free palladium complex that promoted Suzuki–Miyaura cross-couplings between various boronic acids and the *p*-iodobenzyl cysteine residue (chemical incorporation by cysteine alkylation; see **121**, figure 8) of a mutant protein with excellent conversion (greater than 95%) and under mild conditions (pH 8.0, 37 °C). In a subsequent study, they also addressed the issue of non-specific palladium coordination to proteins in Suzuki–Miyaura cross-couplings by using 3-mercaptopropionic acid as a scavenger in order to monitor the conjugation of a *p*-iodophenylalanine-modified maltose binding protein by mass spectrometry [159,160].

In addition to copper and palladium, ruthenium completes the podium of most-employed transition metals for the conjugation of recombinant proteins, essentially in the form of the Hoveyda–Grubbs II catalyst to promote olefin cross-metathesis (CM) of allylic groups-containing UAA **117**–**120** [103,170]. (On a side note, several mentions of ring-opening metathesis polymerization (ROMP) can also be found in the literature regarding protein-polymer conjugation [171]; however, the metathesis step was essentially used to generate polymers and not to conjugate them to proteins, thus falling out of the scope of this review.) Benefitting from the strong allylic chalcogen effect observed in olefin CM, *S*-allylcysteine (Sac) **118** and its selenocysteine analogue (Seac) **120** were identified as excellent substrates for site-selective conjugation of artificial proteins. In general, these UAA were chemically introduced in place of a cysteine residue via direct allylation or transient Dha formation as previously discussed (see **56**, figure 5 and **122**, figure 8), but genetic incorporation of diverse UAA with alkenyl side-chains has also been reported [172,173]. Experimentally, the resulting artificial proteins were conjugated with a wide panel of allylic reagents in rates similar to that of SPAAC for Seac substrates (i.e. approx. 0.3 M^−1^ s^−1^) [174]. Interestingly, addition of magnesium(II) salts to the buffer was found to be critical for successful on-protein olefin CM, in order to prevent unproductive binding of the ruthenium catalyst to the protein surface, as did the use of *t*-BuOH as a co-solvent to facilitate catalyst dissolution [175].

To date, dozens of UAA have been successfully incorporated into proteins—a selection of which is presented in figure 8, listed according to the bioorthogonal technique employed for their functionalization—almost exclusively by genetic code expansion. Using bespoke reagents, bioorthogonal and site-selective conjugation of these artificial proteins has been made possible in live cells, highlighting the strong advantages of these techniques. While chemical strategies for the modification of UAA are plentiful, peptide and protein tags offer a complementary and interesting alternative as they open the door to enzyme-mediated, site-selective conjugation of artificial proteins.

#### 3.1.3. Incorporating enzymatic recognition sequences

Spurred by the powerful applications of bioorthogonal conjugation of UAA-containing artificial proteins, researchers also investigated the genetic engineering of fusion proteins, i.e. chimeric proteins resulting from the assembly of a POI with a peptide or a protein tag that can be covalently and selectively labelled with a small molecule. This selectivity usually comes from the repurposing of a known biological activity of the protein tags, which are essentially derived from enzymes in the context of bioconjugation. The usual method for producing these fusion proteins is via the construction of a corresponding recombinant plasmid, then expressed in different types of cells, thus offering access to live cell labelling applications.

**Figure 9.**
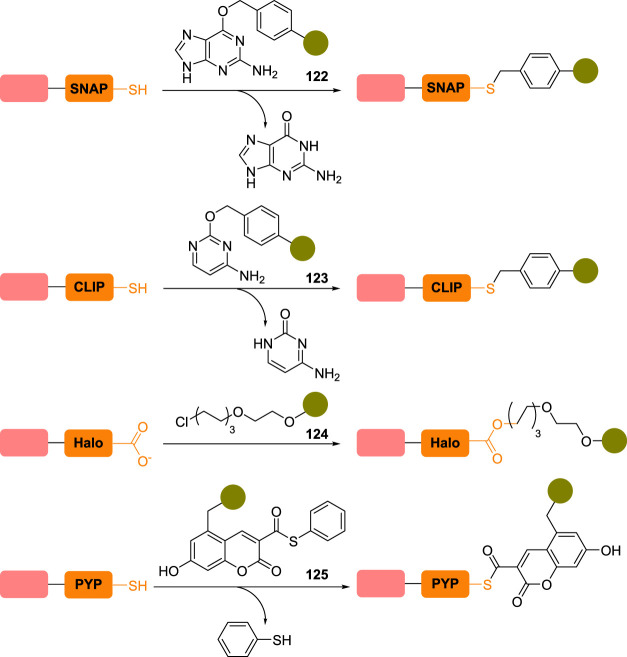
Use of protein tags for the site-selective conjugation of chimeric proteins.

As a prime example, the group led by Kai Johnsson reported in 2003 the use of a mutant enzyme—^W160^hAGT—derived from the human *O*^6^-alkylguanine-DNA alkyltransferase (hAGT), a DNA repair protein that acts as a scavenger of *O*^6^-alkylated guanine nucleobase by transferring the alkyl group to one of its reactive cysteine residues (figure 9) [176]. Taking advantage of this transformation, *O*^6^-benzylguanine derivatives **122** were designed to label rapidly (rate constants up to 10^4^ M^−1^ s^−1^) and selectively engineered hAGT and fusion proteins thereof, leading to a plethora of applications, including *in vivo*, under the trade name SNAP-tag [177]. In the same vein, the CLIP-tag was also developed, which could be functionalized with *O*-benzylcytosine derivatives **123** on a reactive cysteine residue [178]. Interestingly, despite similar structural features, SNAP-tag and CLIP-tag were shown to be orthogonal to one another, allowing the simultaneous and selective labelling of two different fusion proteins.

Switching to another family of fusion proteins, the Halo tag was developed based on *Rhodococcus* haloalkane dehalogenase (DhaA), a bacterial enzyme possessing a reactive aspartate residue (D106) that could be selectively labelled with chloroalkane substrates **124** [179]. Carefully designed mutants were produced, which helped to improve the recombinant enzyme's binding rate (up to a whopping 10^6^ M^−1^ s^−1^) and led to hydrolytically stable conjugates. Bacterial enzymes are also a source of inspiration for the development of other fusion proteins: the photoactive yellow protein (PYP) tag [180,181], which can be selectively functionalized on its reactive cysteine residue C69 by *trans-*thioesterification of coumarin derivatives **125**, or non-catalytic β-lactamases that can be site-selectively acylated on the serine residue S70 [182].

Although the use of fusion protein proved to be a very powerful strategy (e.g. fast labelling kinetics, site-selective conjugation, easy access to structurally diverse payloads, real-time analysis of fast biological processes in living cells), the molecular weight of these chimeric assemblies appeared to be too high in certain cases and altered some key properties of the POI (e.g. structure, function, dynamics, localization) [104].

**Figure 10.**
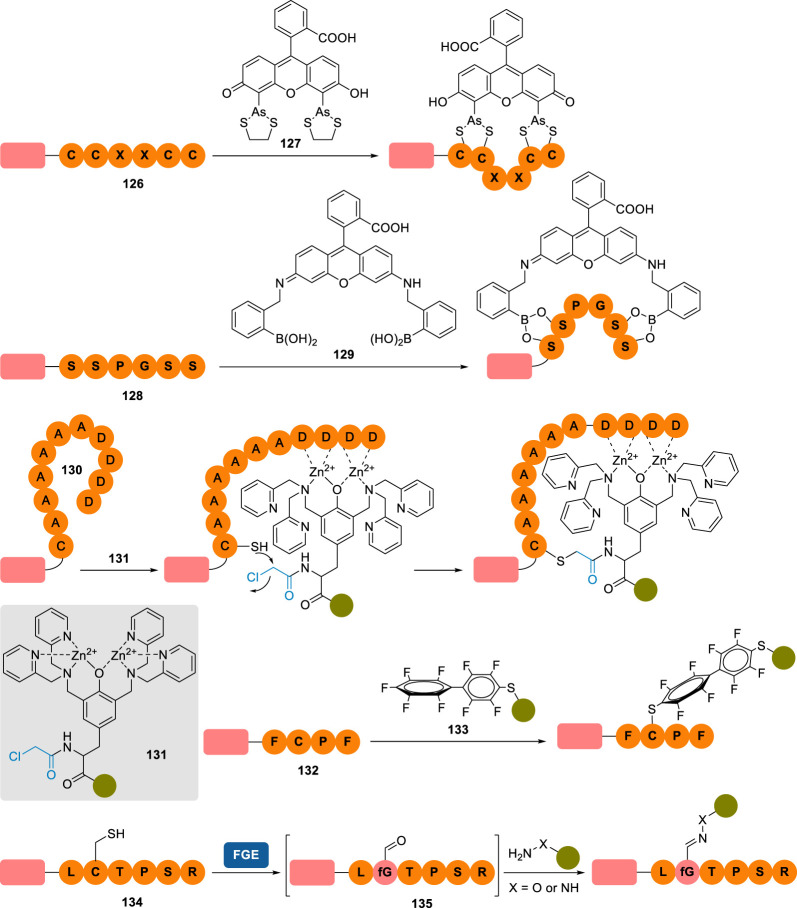
Installation of small peptide tags on the *C*-terminus of a protein of interest and their selective labelling with bespoke probes.

To bypass this issue, shorter peptide tags (0.6–6 kDa) were also designed in parallel and installed on the *N*- or *C*-terminus of the POI (figure 10). It was thought that their smaller size would limit their detrimental impact on the protein's structure and function [104]. In a seminal work, Tsien and co-workers [183,184] reported a tetracysteine tag **126** (CCXXCC; X = any proteinogenic amino acid) able to bind bis-arsenic fluorophores **127** with high selectivity and affinity. Based on a similar design, the tetraserine tag SSPGSS **128** was developed to react selectively with bis-boronic dyes **129** to form macrocyclic boronates [185]. Unlike tetracysteine tag **126**, whose motif is absent in native proteins, the SSPGSS sequence can, however, be found in more than 100 native human proteins, thus affecting the protein selectivity level of this tag in live cells. In 2007, Hamachi and co-workers produced the undecapeptide tag CysAla_6_Asp_4_ (CA_6_D_4_) **130** in which the tetra-aspartate motif can selectively coordinate a zinc(II)-containing electrophilic probe of general structure **131**, helping to direct its reactive α-chloroacetyl motif (highlighted in blue) close to the tag's cysteine residue, resulting in a site-selective alkylation of the peptide tag. Similarly, polyhistidine tags have been employed to complex a tosylate-equipped nickel(II) probe to direct the selective alkylation of one of the histidine tag imidazole, but metal cation-free methods employing similar tags have also been reported [186,187]. More recently, Pentelute and co-workers [188] reported the shortest reactive tag sequence to date, the *C*-terminal FCPF tetrapeptide **132** functioning as a π-clamp and allowing the selective labelling of the tag's cysteine with perfluoraromatic reagents **133**. It is worth stressing that the vast majority of these conjugation reactions occur under mild conditions—a prerequisite when working with live cells—and in minutes to hours.

Albeit falling slightly out of the scope of this review, one can also say a few words about the modification of peptide tags by formylglycine-generating enzyme (FGE). In eukaryotes, FGE in combination with a copper cofactor catalyses the conversion of a cysteine residue to an aldehyde-containing formylglycine (fGly) residue in the minimal consensus sequence CXPXR (X = any proteinogenic amino acid but proline) of sulfatases [189,190]. Taking advantage of the promiscuity of FGE, the incorporation of the aldehyde tag CXPXR—usually in the form of the hexapeptide LCTPSR **134**, the wild-type sequence found in human type I sulfatases—in various fusion proteins, including antibodies, allowed the smooth FGE-catalysed installation of fGly residues (see **135**, figure 10), which were then selectively functionalized via oxime or hydrazone ligation [191–193]. Unlike enzyme-mediated bioconjugation techniques (e.g. using sortase A, transglutaminases, protein farnesyltransferase), which will not be discussed in this review, the enzyme in this particular case only *assists* in the conjugation of the POI by converting it to a transient, reactive electrophile but does not *participate* in its subsequent derivatization, making conjugation a well and truly chemical step.

Thus, site-selective chemical conjugation of artificial proteins offered prominent results thanks to the incorporation of natural or UAA, either as single residues or as part of peptide and protein tags. However, generation of such proteins can be a costly and time-consuming process. Coupled with a restricted access to only a certain degree of conjugation (classically from 1 to 2) and potential immunogenicity issues due to the artificial incorporation of amino acids or peptide sequences in the POI, bioconjugates of artificial proteins present some limitations that prevent their widespread use, undermining their panacea-like image. While far more challenging chemically speaking, site-selective methods for the conjugation of native proteins can be seen as a way to overcome these limitations.

### 3.2. Site-selective labelling of native proteins

As an alternative to artificial proteins, the development of site-selective strategies for the conjugation of native proteins has emerged over the past few years. Compelling results have been obtained *in vitro*, in complex biological environment, notably thanks to ligand-directed proximity-driven conjugation [194]. However, these would not be discussed in the following sections, as the observed site-selectivity originates from a preliminary molecular recognition between a protein and its ligand and not from the reactive chemical component itself. Thus, the chemical strategies about to be discussed aim to be applicable to a broad range of native proteins, and have been designed to target various types of amino acids. Unsurprisingly, the most abundant lysine has been one the most studied residues.

#### 3.2.1. Lysine residues

**Figure 11.**
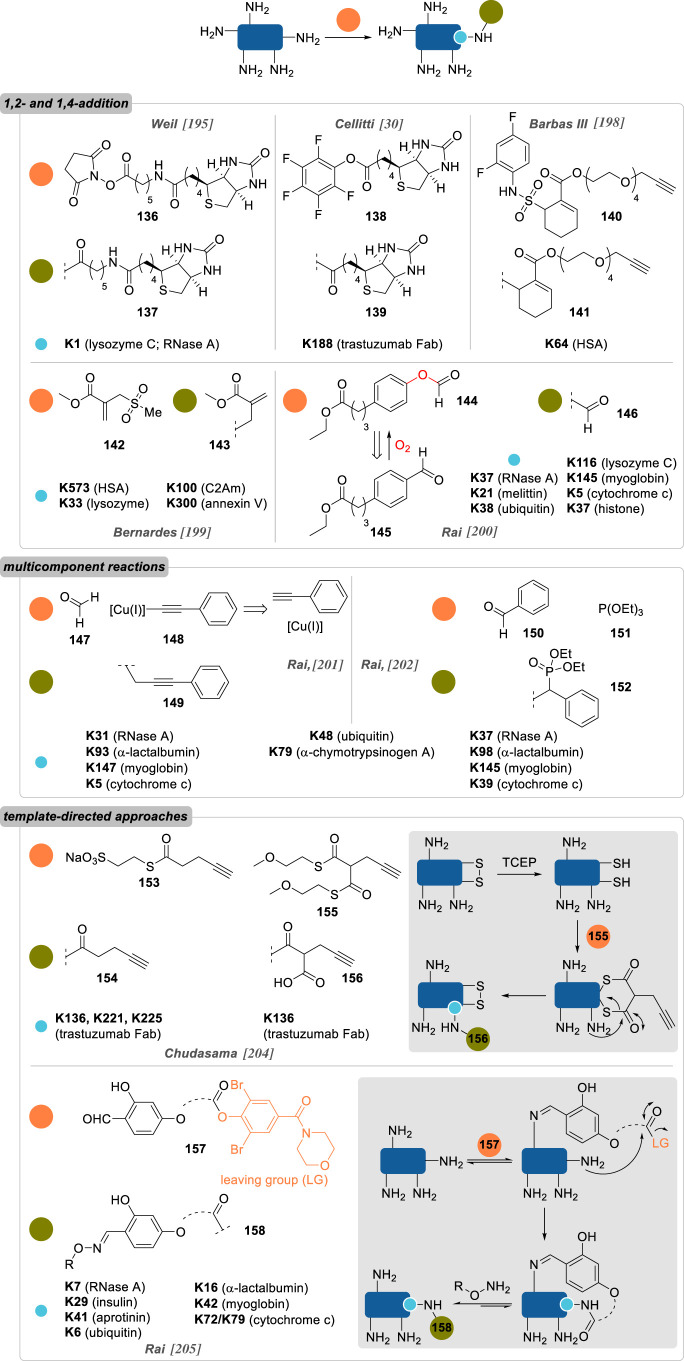
Overview of site-selective chemical conjugation of native proteins on lysine residues (R, alkyl chain with payloads of interest; TCEP, *tris*(2-carboxyethyl)phosphine).

As previously mentioned, lysines are both abundant and surface-exposed in the vast majority of proteins, thus complicating their site-selective conjugation. However, some approaches have been proposed to tackle this challenge on native proteins, relying on kinetic control, bespoke electrophilic reagents, multicomponent reactions (MCR) or template-directed approaches (figure 11).

One of the most common strategies for the chemoselective modification of lysines is based on the use of activated esters (see §2.2.1.). In 2012, Weil and co-workers [195] showed that a site-selective variation of this approach was possible, with the successful modification of RNase A and lysozyme C via a kinetically controlled labelling. Portionwise addition of sub-stoichiometric amounts of NHS-biotin derivative **136**—i.e. 0.005 equivalent per minute, over 100 min—led to the modification of a single lysine residue on the POI, K1 in both cases, which was explained by stereoelectronic effects: best solvent accessibility in the case of RNase A; hydrogen bond between K1 side-chain's ammonium and a neighbouring carboxylate group, increasing K1 nucleophilic character in lysozyme C. As expected from the conjugation conditions, site-selectivity occurred to the detriment of partial conversion and limited yield, even though this could be overcome by affinity purification on avidin column of the biotin-containing monoconjugates.

Moving on to bigger proteins, Cellitti and co-workers [30] studied the single labelling of K188 on the Fab fragment of the monoclonal antibody trastuzumab (50 kDa). Building on a previously published patent reporting the use of pentafluorophenyl esters **138** to achieve this single residue conjugation (up to 70% labelling) [196], they showed that lowering the temperature to 4 °C or using flow chemistry to perform the conjugation helped to increase the yield and reach nearly quantitative labelling of this residue. Interestingly, site-directed mutagenesis studies highlighted the extreme importance of two neighbouring residues, H189 and D151, whose mutation to alanine residues led to little to no conjugation of the resulting mutants. While it was hypothesized that the single labelling of K188 was explained by the formation of a triad charge-relay system, no definitive mechanism was proposed by the authors.

The fact that the local microenvironment of a residue can influence its reactivity is at the basis of how enzymes exert their biological role. In the case of lysine, it has been found that ε-amino groups located at internal positions in proteins could have p*K*_a_ values as low as 5.3, more than 5 units lower than the classical 10.4 value observed in water [197]. Being less prone to being protonated at physiological pH, such residues represent an attractive target in the context of site-selective conjugation, leading to the development of tailored reagents.

Barbas and co-workers [198] showed that α,β-unsaturated sulfonamides **140**—and their CuAAC-functionalized derivatives—could selectively modify the K64 residue of human serum albumin (HSA) in human plasma, leading to exceedingly stable adducts *in vitro* (*t*_1/2_ > 28 d in human plasma at 37 °C) and *in vivo* (*t*_1/2_ ∼ 40 h in BALB/c mice). The influence of HSA's tertiary structure on the reactivity of K64 was highlighted by an absence of reaction between **140** and the simple lysine amino acid, even though no further theory was proposed to explain this observation.

Influence of local microenvironments on α,β-unsaturated sulfonamides reactivity and lysine conjugation was further supported by Bernardes and co-workers [199] a few years later. In a seminal work published in 2018, they reported the use of sulfonyl acrylate reagent **142**, reminiscent of Barbas' sulfonamide **140**, for the site-selective modification of the most reactive lysine residue in various proteins. It was anticipated that lysines' ε-amino groups with the lowest p*K*_a_—due to their location in a rather hydrophobic microenvironment—would be the most nucleophilic and could react selectively with **142** via a chair-like H-bonded transition state. Comparison between theoretical predictions of p*K*_a_ and peptide mapping analyses run on a handful of monoconjugated proteins seemed to corroborate this postulate: K573 (theoretical p*K*_a_ 10.39) was identified as the sole conjugation site in HSA, K33 (th. p*K*_a_ 9.48) in lysozyme, K100 (th. p*K*_a_ 10.13) in the C2A domain of Synaptotagmin-I (C2Am) and K300 (th. p*K*_a_ 10.35) in Annexin V. Interestingly, these findings contrast with that of Barbas (*vide supra*) and Rai (*vide infra*), who identified K64 in HSA and K116 in lysozyme as the sole conjugated lysine residue, respectively, showing that the reagent structure has a strong influence in the site-selectivity of the conjugation, and not only the residue's accessibility and p*K*_a_ value.

Taking advantage of the *in situ* generation of formate ester **144** from the oxygen-mediated oxidation of benzaldehyde **145**, Rai and co-workers [200] reported the chemoselective and site-selective formylation of various proteins: RNase A (K37), melittin (K21), ubiquitin (K38), lysozyme C (K116), myoglobin (K145), cytochrome c (K5, albeit *N*-terminus acetylation was necessary in this case to achieve site-selective conjugation) and histone (K37), with moderate to good conversion (50–82% after 24–48 h of reaction). Interestingly, all formylated lysines were found to be located in ordered secondary structures (i.e. α-helix, loop, β-sheet) and not necessarily the most solvent-accessible ones.

Drifting away from the classical use of reactive electrophiles, the same group also opted to investigate MCR for the site-selective conjugation of lysines. Using either formaldehyde **147** or benzaldehyde derivatives **150**, ε-amino groups were first converted to latent imines, which reacted in a subsequent step with carefully selected nucleophiles—either *in situ* formed copper(I) phenylacetylide **148** (A^3^ coupling) [201] or triethylphopshite **151** (phospha-Mannich) [202], respectively—with excellent chemo- and site-selectivity. Of the various proteins evaluated in both studies, some were shown to be labelled on the same lysine residue, irrespective of the MCR employed under identical reaction conditions: ubiquitin (on K48) and α-chymotrypsinogen A (K79). The majority, however, showed dissimilar conjugation sites depending on the strategy employed—RNase A (phospha-Mannich: K37; A^3^ coupling: K31), α-lactalbumin (phospha-Mannich: K98; A^3^ coupling: K93), cytochrome C (phospha-Mannich: K39; A^3^ coupling: K5), myoglobin (phospha-Mannich: K145; A^3^ coupling: K147)—stressing even more the prevalence of the reagent structure on the conjugation site.

As proposed by Gothelf and co-workers in 2014, another strategy for the site-selective conjugation of native proteins employs template-directed approaches. They took advantage of histidine-rich regions in different proteins to form transient metal complexes with divalent cations, helping to direct an NHS ester equipped with a *tris*-nitrilotriacetic acid chelating handle to label only a handful of neighbouring lysines [203]. This idea of using another amino acid as a temporary anchor point to direct—via electrostatic interactions or covalent bond formation—the modification of a neighbouring lysine residue can also be found in subsequent studies. Chudasama and co-workers [204] showed that cysteines could act as valuable relays for the site-selective modification of lysines on the Fab fragment of trastuzumab with thioesters **153** and **155**, in a strategy reminiscent of native chemical ligation (figure 11). After reduction of the disulfide bond of the Fab fragment, a first *trans*-thioesterification between the cysteine thiol and a thioester was followed with a ‘cysteine-to-lysine transfer’ (i.e. *S*,*N*-acyl migration) on a proximal lysine. While this first approach could not be coined site-selectively *per se*, with three labelled lysines in the vicinity of the disulfide bond being identified (K136, K221 and K225), the use of bis-thioester **155** for the formation of an intermediate bridged conjugate led to the sole conjugation of K136 (80% conversion), presumably thanks to conformational effects.

More recently, Rai and co-workers [205] pursued their effort toward site-selective conjugation of native proteins and described a lysine-directed lysine modification method. They designed a library of reagents of general structure **157** bearing two reactive groups, an aldehyde and a phenol ester, separated by a spacer of variable length. The aldehyde first led to the rapid but reversible formation of imines with multiple solvent-accessible lysines, helping to direct the intramolecular acylation of a neighbouring lysine by the phenol ester in a slow but irreversible second step. Addition of an *O*-alkoxyamine solution in a final step permits the removal of all remaining imines while functionalizing the POI in the form of conjugates **158**—a procedure that also gave the possibility of trapping and isolating pure mono-labelled conjugates on hydrazide-activated resins before a trans-tagging release with *O*-alkoxyamine probes. Site-selectivity was achieved on different proteins by varying the chemical structure of the spacer: RNase A was selectively modified on K7 (34% conversion), insulin on K29 (48%), aprotinin on K41 (86%), ubiquitin on K6 (35%), α-lactalbumin on K16 (37%) and myoglobin on K42 (26%). Interestingly, varying the spacer also led to different labelling sites on cytochrome C, either K72 or K79 (33% versus 28% conversion, respectively), offering a valuable modularity to this strategy.

Compelling results have thus been obtained on a panel of native proteins of diverse molecular weights—from as low as 2.8 kDa to greater than 66 kDa—via different approaches. As is apparent from these studies, the microenvironment of the lysine and the chemical structure of the electrophile are both equally important, and the slightest variation in one or the other parameters can drastically alter the site-selectivity of the strategy. While labelled lysine residues were systematically identified by peptide mapping studies in almost all studies, difficulties in identifying the precise conjugation site seem to have emerged with full antibodies, possibly because of their high molecular weight and their numerous lysines. Focusing instead on cysteine conjugation for full antibodies helped to overcome this limitation.

#### 3.2.2. Cysteine and cystine residues

As previously mentioned, cysteines with free side-chain thiols are relatively scarce in nature, forming usually homo- or hetero-dimers via oxidized disulfide bonds, such as the cystine residues bridging heavy and light chains in antibodies. While reduction of the inter-chain disulfide bonds of the most abundant IgG1 can give access to eight free thiols, their chemoselective labelling can have a detrimental impact on the structure, shape, stability and activity of the protein, while still leading to heterogeneous mixtures of conjugates due to incomplete conversion [206]. Thus, chemical methods to conjugate covalently free cysteines ‘in pairs’, effectively rebridging the disulfide bond and cross-linking the protein, has been proposed as a way to address this issue [207].

**Figure 12.**
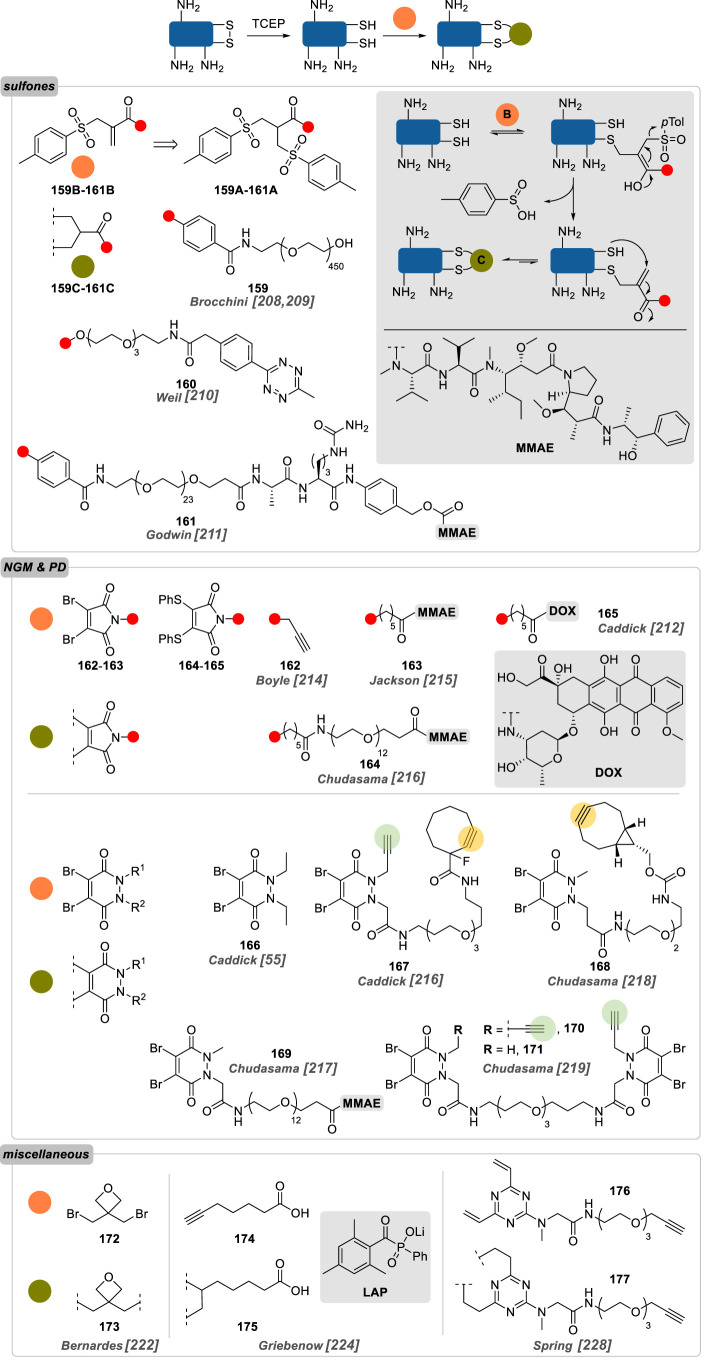
Overview of site-selective chemical conjugation of native proteins on cystine (TCEP, *tris*(2-carboxyethyl) phosphine; coloured circles on compounds **167**, **168**, **170** and **171** indicate functionalization sites as detailed in figure 7).

Brocchini and co-workers [208,209] were the first ones to reconstruct covalent bonds between two reduced cysteines in proteins by forming a three-carbon bond between the two thiols with monosulfone cross-linking reagents **159B** deriving from bissulfone precursor **159A** by simple incubation at pH 7.8 (figure 12). After disulfide reduction, a successive thio-Michael addition/sulfinic acid elimination/second thio-Michael addition (see mechanism in sidebar) allowed the facile PEGylation of interferon α-2b, as well as l-asparaginase and the Fab fragment of the monoclonal antibody Q4120/ADP318, without any apparent disulfide scrambling. Building on this work, monosulfone reagent **160B** was successfully applied as a rebridging reagent for the production of homogeneous trastuzumab Fab fragment conjugates [210]. The presence of a tetrazine handle allowed facile functionalization of the resulting conjugates with *trans*-cyclooctene-equipped payloads (i.e. fluorophores and short PEG).

Applied to the intact antibody, MMAE-sulfone reagent **161B** led to the favoured formation of a DAR4 conjugate (78%), showing both *in vitro* and *in vivo* targeted cytotoxicity [211].

Next-generation maleimides (NGM), such as dibromomaleimides **162** and **163**, and dithiophenolmaleimides **164** and **165**, were also employed as rebridging reagents of disulfide bonds [212–215]. The incorporation of two nucleofuges across the maleimide double bond allowed the consecutive addition of two thiols via a similar addition–elimination–addition sequence as previously described for sulfones **159B–161B**. These reagents helped construct numerous ADCs, some with improved properties over conventional maleimide-conjugated species in terms of efficacy, toxicity and pharmacokinetics. Excellent yields and conversion have been reported, with DAR 4 being the sole immunoconjugates detected in certain cases, albeit concurrent generation of half-antibody isoforms via intra-chain bridging and disulfide scrambling could systematically be observed.

In 2011, Caddick and co-workers [55] reported the conjugation of proteins and the bridging of cyclic peptides with dibromopyridazinedione **166** (PD). While being structurally related to NGM, PD possess the advantage of presenting four points of attachment: the two sp^2^ carbons of the alkene moiety, reacting chemoselectively with free thiols through two consecutive addition–elimination steps, and the two pyridazine nitrogens, grafted with different bioorthogonal tags allowing the dual labelling of the POI. A fewyears later, Caddick and co-workers [216] reported the use of PD **167** for the reconstruction of inter-chain bonds of the full antibody trastuzumab. Since the reagent was designed to bear two orthogonal handles, a strained and a terminal alkyne, the rebridged antibody could be further functionalized via successive SPAAC and CuAAC, yielding immunoconjugates with two different payloads per reconstructed disulfide bond.

Based on these results, new PD reagents were also designed to afford homogeneous antibody conjugates with lower DAR. DAR4 conjugates were obtained by only adding a single functionalization site on PD, like the strained alkyne in PD **168** or the drug MMAE in PD **169**, resulting in the generation of a panel of PD with very effective rebridging abilities—less than 5% of half-antibody was generally detected, and homogeneous DAR4 ADC could be produced in excellent yield [217,218]. Connecting two PD together with a PEG linker—whose length was critical for the successful outcome of the strategy—allowed the simultaneous rebridging of two disulfides on trastuzumab, while the incorporation of one (**171**) or two (**170**) terminal alkynes per PD dimer offered a selective access to DAR2 or DAR4 conjugates, respectively [219]. Further optimization of pyridazinediones led to valuable applications, such as the possibility of conducting disulfide reduction and rebridging in a single step with ‘two-in-one’ reagents [220], or the generation of homogeneous bispecific Fab fragments conjugates [221].

Another rebridging reagent, the dibromo oxetane derivative **172**, was proposed in 2017 by Bernardes and co-workers [222]. Albeit being deprived of a functional handle for further derivatization of the resulting protein conjugates, dibromooxetanes proved to be effective for the stapling of cysteine residues in different proteins, including antibody fragments. Intriguingly, replacing the oxetane motif with an alkene altered the reactivity of the electrophile, leading only to negligible stapling [223].

Thiol-yne reaction was also proposed as a disulfide-rebridging photochemical strategy by Griebenow *et al.* [224] (figure 12). After reduction of the Fab fragment of the antibody M14-G07, the rebridging was carried out under UV light at 365 nm in the presence of 6-heptynoic acid **174** and lithium phenyl-2,4,6-trimethylbenzoylphosphinate (LAP) as radical initiator. While the presence of covalently disulfide-stapled Fab was demonstrated, conversion was poor (40%) and the reaction necessitated elevated antibody concentrations to be effective (46.1 mg ml^−1^).

Structurally related to this strategy, addition to electron-deficient alkenes—such as divinyl triazine **176**—and alkynes was also explored by several groups, including ours, developing various rebridging reagents that were tested on native proteins—mostly full antibodies [225–228]. While potent ADCs could be produced in this way, disulfide scrambling was systematically observed, as evidenced by the formation of half-antibodies in all cases.

Cysteines rebridging reagents have thus offered access to site-selectively conjugated native proteins in excellent yields. NGM and PD have been the most studied reagents in this regard, and were shown to be the most effective at minimizing disulfide scrambling. More interestingly, varying their structure permitted a controlled access to DAR8, 4 and 2 ADCs.

While lysine and cysteine take most of the light, other residues have also been investigated as valuable targets for accessing site-selective conjugation of native proteins. With a lower p*K*_a_ than the ε-amino side-chain of lysine, α-amino groups of *N*-terminus possess a peculiar reactivity of particular interest in this context, explaining why it has gained increasing attention in recent years.

#### 3.2.3. *N*-terminal residues

Numerous reports about general strategies for the chemo- and site-selective modification of α-amino groups of native proteins can already be found, including in the recent literature [229]. Most of these methods employ carbonyls, and aromatic aldehydes in particular, but sulfonamides, phthalimides or enzymes were also demonstrated to be valuable candidates for this job. Pushing the constraints even further, focusing on the conjugation of particular *N*-terminal amino acids—notably *N*-terminal cysteine, glycine and proline—offers an extra level of selectivity.

**Figure 13.**
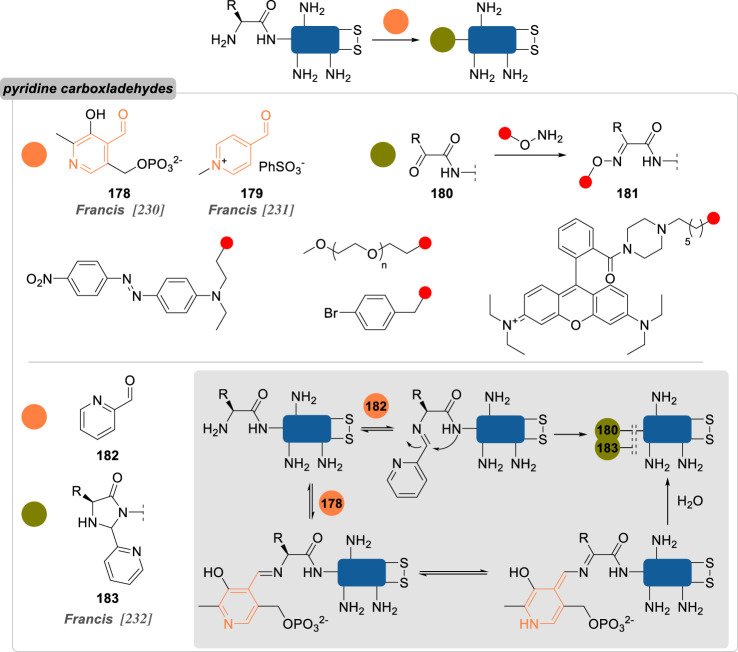
*N*-terminus selective reagents based on pyridine carboxaldehyde scaffolds (R, side-chain of proteinogenic α-amino acids).

In 2006, Francis and co-workers [230] were the first to report the use of pyridine carboxaldehydes for the selective oxidation of the *N*-terminus (figure 13). Using pyridoxal-5-phosphate (PLP) **178**, *N*-terminal α-amino acid residues were converted to pyruvamides **180** and further derivatized to oximes **181** equipped with various payloads. *N*-terminal valine, glycine, lysine, methionine and aspartate residues of several native proteins were successfully modified, with conversions up to 80%, but it was anticipated that other residues would not be suited for this approach: serine, threonine or cysteine due to possible oxazolidine/thiazolidine ring formation; tryptophan, because of competing Pictet–Spengler cyclization; and proline, due to a lack of reactivity. Alternatively, in 2013, they identified *N*-methylpyridinium-4-carboxyaldehyde **179** (Rapoport's salt) as a new and more effective reagent, proceeding via the same mechanism, and applied it to the selective labelling of the heavy-chains *N*-terminal glutamates of the antibody trastuzumab (67% conversion), while the light chains *N*-terminal aspartate were left unlabelled [231]. As the scope of *N*-terminal α-amino acids was found to be limited with these reagents, 2-pyridinecarboxyaldehyde (2-PCA) **182** was later reported as a more broadly applicable strategy for the site-selective conjugation of peptides and proteins, irrespective of the nature of the *N*-terminus [232]. Proceeding via another mechanism, this reagent led to the formation of imidazolidinones **183**, with the imine formed in a first step undergoing intramolecular addition of the *n* + 1 α-amino group in a second step—explaining why proteins with proline as the *n* + 1 residue failed to be conjugated via this strategy. While this conjugation proved to be partially reversible at 37 °C, it benefitted from a large protein scope, with good conversions being observed in most cases. Even though *N*-terminal serine-containing proteins were listed as potential limitations to the PLP strategy (*vide supra*), thioredoxin was still successfully labelled with 2-PCA (63% conversion) and the potentially competing oxazolidine formation not discussed by the authors.

**Figure 14.**
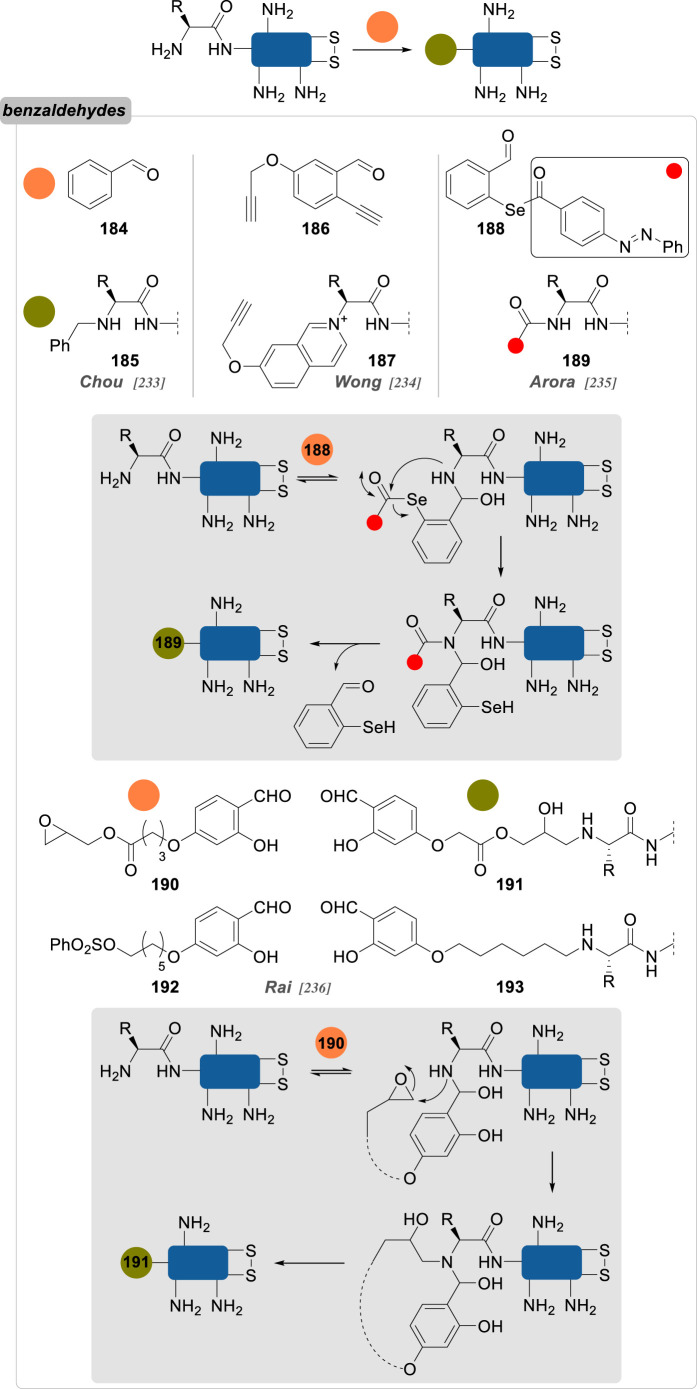
*N*-terminus selective reagents based on benzaldehyde derivatives (R, side-chain of proteinogenic α-amino acids).

Further evidence for an absence of oxazolidine ring formation when *N*-terminal serine is reacted with aromatic aldehydes has been given by Chou and co-workers [233], who conducted the reductive amination of six native proteins and different nonapeptides varying only by their *N*-terminal residues (figure 14). With benzaldehyde **184** in the presence of sodium cyanoborohydride, nearly quantitative conversions to the *N*-terminal alkylated peptides were observed in all cases with the exception of cysteine, where thiazolidine ring formation rivalled that of reductive amination. Interestingly, with 2-PCA, only alkylation was observed and not imidazolidinone formation as previously described by Francis, which could be explained by slightly different reaction conditions between the two studies—different substrates, different pH—but also by kinetic/thermodynamic considerations, imidazolidinone ring formation being reversible.

Taking inspiration from the previous strategies developed by Francis and co-workers, Wong and co-workers [234] recently reported 2-ethynylbenzaldehyde **186** (2-EBA) as a new *N*-terminus conjugation reagent. A cascade starting with imine formation followed by a 6-*endo-dig* cyclization delivered an isoquinolinium derivative as the final product **187** and was used to label peptides and proteins. In the latter case, conversions were found to be modest for all three proteins evaluated—lysozyme, RNAse A and BCarg mutant—and competition between *N*-terminal α-amines and lysine side-chain ε-amine could systematically be observed for conversions over 10%. Side reactions competing with isoquinolinium cyclization after imine formation—e.g. oxazolidine, thiazolidine, imidazolidinone—were not evidenced, suggesting again the profound influence of the aldehyde stereoelectronic effect on the reactivity of its α-imine derivatives.

Another strategy making use of aldehyde reactivity and so-called aldehyde capture ligation (ACL) was described in 2015 by Arora and co-workers [235]. In a mechanism reminiscent of that of native chemical ligation, the ACL employs benzeneselenoesters **188** as acylating reagents, whose reactivity is enhanced by a neighbouring *o*-aldehyde group—up to 100-fold compared to the unsubstituted benzeneselenoester analogue—helping to promote hemiaminal/imine formation and direct the subsequent *Se*-to-*N* intramolecular acyl shift (see mechanism, figure 14). Site-selective conjugation of ubiquitin could be obtained with just five equivalents of **188**, albeit 40 h were necessary to attain good conversion.

This idea that a fast and reversible first reaction, such as imine formation, can accelerate the kinetics of a second irreversible reaction and thus confer a certain degree of site-selectivity to the overall transformation can be found in the work of Rai and co-workers [236], who showed in 2018 that *N*-terminus selective modification of RNase A and aprotinin could be achieved with epoxides and sulfonate esters *only* if they were linked to a benzaldehyde handle in a bifunctional reagent such as **190** and **192**, respectively. By contrast to their subsequent work in 2020 in which they used a similar approach to selectively label a single lysine or histidine residue (see **157** and **158**, figure 11; and **240**–**242**, figure 18), in this case it is the same nitrogen—the α-amino group—that acts as both the directing group and the labelling site, with the transient imine formed in the first step being selectively and irreversibly alkylated in the second. Such a difference in reactivity pattern is remarkable given the great similarity between these two approaches (both in terms of the structure of the reagents and the reaction conditions employed) and further emphasizes the sensitivity of site-selective chemical conjugation techniques to the slightest variations.

While aromatic aldehydes have been shown to be privileged reagents for the *N*-terminus selective conjugation of native proteins, several other electrophiles have also been reported.

**Figure 15.**
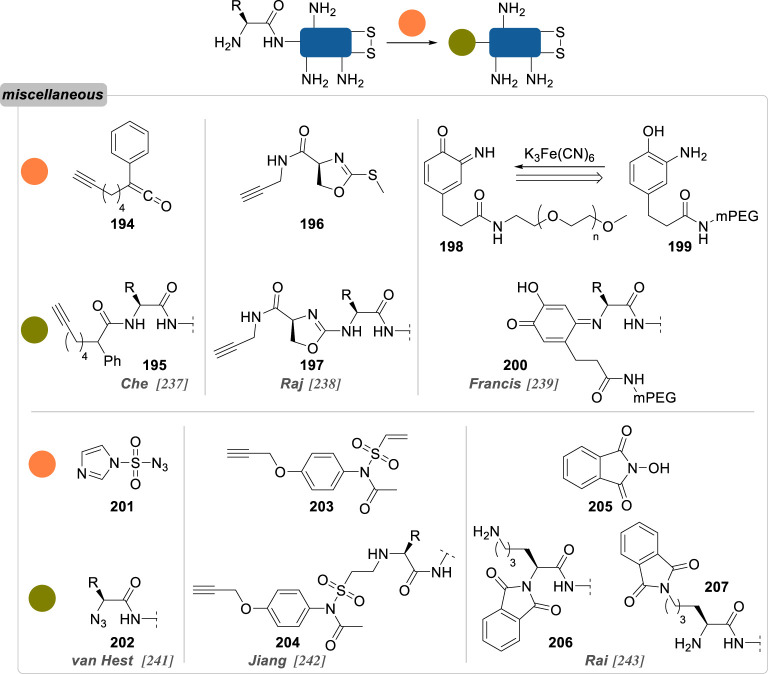
*N*-terminus selective reagents based on miscellaneous derivatives (R, side-chain of proteinogenic α-amino acids).

Ketenes **194** were found to selectively modify the *N*-terminus of insulin, lysozyme, RNaseA and BCArg, even though low conversions were generally observed, as well as traces of di- and tri-modified conjugates (figure 15) [237].

2-Methylthio oxazolines **196** have also been applied to the site-selective conjugation of native proteins with almost complete conversion in several cases [238]. In addition, the oxazoline group helped to increase ionization of peptide fragments by mass spectrometry and enhance the sensitivity of the method, making this conjugation strategy valuable for proteomics studies even though it was only limited to relatively low molecular-weight proteins (i.e. less than or equal to 17 kDa).

Francis and co-workers [239] proposed an oxidative strategy for the efficient labelling of α-amino groups. Relying on the *in situ* chemical oxidation (using potassium ferricyanide) of *o*-aminophenols **199** to reactive *o*-iminoquinone intermediates **198**, formation of *o*-quinone adducts **200** occurred selectively on *N*-terminus. Moderate conversions were obtained on various native proteins, presumably because of the very nature of their *N*-terminal residue or their solvent accessibility: MS2 (*N*-ter Ala; 0%), lysozyme (*N*-ter Ala; 38%), RNase A (*N*-ter Lys; 30%), myoglobin (*N*-ter Gly; 34%) and chymotrypsinogen (*N*-ter Cys; 42%). Interestingly, *N*-terminal proline residues seemed to be favoured substrates in this transformation, as exemplified by a mutant *N*-ter-proline GFP leading to better conversion (61%) than its wild-type, *N*-ter-Met analogue (24%) under the same conjugation conditions.

In an effort to improve the key oxidation step of phenol derivatives, the same group later reported the use of tyrosinase to convert benchstable 4-substituted phenol reagents to their corresponding *o*-quinone analogues, a reaction that potassium ferricyanide failed to promote [240]. The resulting quinones gave excellent *N*-ter-proline selectivity and high yields, even though small amounts of double modification were consistently observed on several proteins and under various reaction conditions. Importantly, quinone payloads were shown to be reactive toward alkoxyamines and free thiols, presumably because of oxime formation and thio-Michael addition, a potential advantage for the introduction of different payloads post-conjugation that has not been exploited further by the authors.

A handful of reports also detail the use of sulfonyl-based reagents for *N*-terminus selective conjugation. Imidazole-1-sulfonyl azide **201** was used by van Hest and co-workers [241] as a convenient way to convert amines to azides on proteins via diazo transfer. *N*-terminus selectivity was demonstrated on two elastin-like polypeptides and on CalB, even though the presence of minute amounts of ε-azidation side reaction could consistently be detected. Intriguingly, lysozyme led only to lysine conjugation, presumably because of the poor solvent accessibility of *N*-terminus.

While vinyl sulfones were shown to be cysteine-selective reagents (see §2.2.2., compound **36**), vinyl sulfonamide **203** was successfully employed for the site-selective conjugation of α-amino groups of somatostatin, lysozyme and RNase [242]. Mildly acidic conditions (i.e. pH 6.0) offered excellent site-selectivity, with only trace amounts of double conjugation observed, even though conversions were rather low, with unconjugated proteins being the most abundant species in the cases of lysozyme and RNase A.

Finally, phthalimidation of primary amines was also found to be effective for the residue-selective modification of RNase A [243]. Using a single equivalent of *N-*hydroxyphthalimide **205** led to the sole conjugation of K1 (50% conversion), even though clear evidence that α-phthalimidation (**206**) occurred preferentially over ε-phthalimidation (**207**) was not given.

Increasing the level of selectivity further, some strategies recently surfaced focusing on the conjugation of some specific *N*-terminal residue, taking advantage of a peculiar reactivity caused by structural aspects (e.g. the C*_α_*-unsubstituted glycine, the secondary amine of proline) or by the presence of a complementary reactive group on the side-chain.

**Figure 16.**
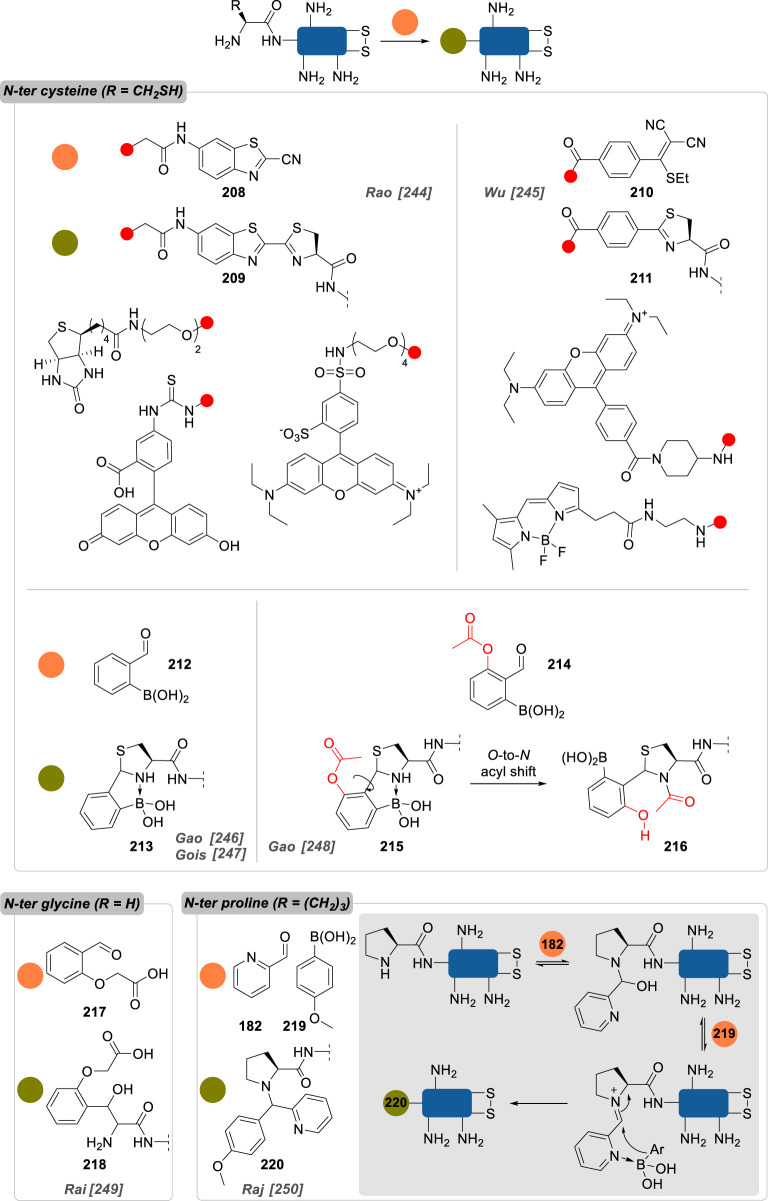
*N*-terminal cysteine, glycine and proline selective strategies based on the formation of 4,5-dihydrothiazole/thioazolidine, 1,2-aminoalcohol and *N*-alkylated conjugates, respectively.

Taking inspiration from the synthesis of d-luciferin, Rao and co-workers [244] reported in 2009 a water-compatible condensation reaction for the selective labelling of 1,2- and 1,3-aminothiols in general, and of *N*-terminal cysteines in particular (figure 16), using 2-cyanobenzothiazoles **208** (CBT). While reversible, this ligation was shown to possess elevated kinetics (greater than 9.0 M^−1^ s^−1^) and was applied to the rapid conjugation of several proteins in live cells in just 30 minutes, thanks to the development of various CBT-containing probes equipped with fluorophores or biotin.

Cyclization of the 1,2-aminothiol motif of *N*-terminal cysteines to 4,5-dihydrothiazole was also employed by Tsai and co-workers in 2020 with 2-((alkylthio)(aryl)methylene)-malononitrile (TAMM) reagents **210** [245]. The reaction proceeded smoothly under similar conditions to those employed by Rao with CBT **208**, and with comparable kinetics, allowing the bioorthogonal conjugation of various peptides and artificial proteins, including in live cells.

Focusing instead on the formation of thioazolidine analogues, Gois and Gao independently described 2-formylphenyl boronic acid **212** (2-FPBA) for the modification of *N*-terminal cysteines at neutral pH of a library of peptides and of the model protein villin headpiece subdomain [246,247]. While thioazolidine formation typically requires harsh conditions and prolonged reaction times, the presence of a boronic acid helps accelerate its formation—as demonstrated by second order rate constants comprised between 10^2^ and 10^3^ M^−1^ s^−1^ and reactions necessitating only a few minutes to reach completion—and stabilize the resulting heterocycle via the formation of an N–B dative bond. However, this stabilization is only partial, as thiazolidine adducts were shown to dissociate in the presence of free cysteine, hydroxylamine derivatives or under mildly acidic pH.

To address this limitation, Gao and co-workers [248] hypothesized that the acylation of the thiazolidine nitrogen would prevent imine reformation and ring-opening, and thus lead to more stable conjugates. Developing boronic acid reagents **214** based on an acetylated salicaldehyde motif, they managed to observe formation of the expected adducts **216**, with the *O*-to-*N-*acetyl shift from boron adduct **215** being the rate limiting step. Complete conversions were typically observed within 2 h at pH 6.0 with various peptides and proteins, and the strategy was also applied to the chemical modification of a phage library. As hypothesized, the intramolecular acetyl transfer led to a substantial gain in stability for the thiazolidine adducts, which were shown to be insensitive to the presence of free cysteine, and stable for days at pH 5.0.

Switching to another specific family of *N*-terminal residues, Rai and co-workers [249] showed in 2019 that *N*-terminal glycines could form 1,2-amino alcohols **218** when reacted with *ortho*-substituted benzaldehydes **217** (figure 16). Notwithstanding the excessive amount of reagent employed (500 equivalents), the reaction proceeded under smooth conditions—pH 7.8, 24 h, 25 °C—and proved to be selective for *N-*terminal glycines as proteins exhibiting *C*_α_-substituted *N*-terminal residues were not labelled in competition experiments.

More recently, selective modification of *N*-terminal prolines was also proposed. Francis and co-workers [239,240] used quinone derivatives as conjugation reagents, generated *in situ* via chemical or enzymatic methods (*vide supra*), while Raj and co-workers [250] employed the Petasis reaction for the preferential modification of peptides with an *N*-terminal proline (figure 16). Applied to proteins, a mixture of 2-PCA **182** and *p*-methoxyphenyl boronic acid **219** did not lead to any detectable conjugates when myoglobin (*N*-ter Gly) and α-lactalbumin (*N*-ter Ala) were evaluated, but successfully modified creatine kinase and aldolase (both *N*-ter Pro), even though conversions were not given and no further analyses were conducted to confirm the site-selectivity of the conjugation. Interestingly, formation of imidazolidinone as reported by Francis *et al.* (figure 13) with 3-hydroxy-2-pyridinecarbaldehyde could be suppressed in this case to favour Petasis reaction by finely tuning the reaction conditions and amount of boronic acid nucleophile.

Strategies focusing on *N*-terminus selective modification have thus played on various parameters to produce successful results: the usually observed solvent accessibility of α-amines coupled with their lower p*K*_a_ value compared with ε-amines, but also the presence on the α-carbon of both the protein's amide backbone and relatively acidic protons, both of which could confer a particular reactivity to transient imines formed on these positions. Similarly, the nature of the *N*-terminal residue itself can confer an additional degree of selectivity and a handful of strategies targeting only specific residues have thus been successfully devised, essentially centred on *N*-terminal cysteine, glycine and proline.

In the following paragraph, we will talk about the site-selective conjugation of residues which have not benefited—yet—from extensive research compared with their predecessors; these concern tyrosine, tryptophan, histidine, methionine, arginine, serine, aspartate, glutamate and *C*-terminal residues.

#### 3.2.4. Miscellaneous

Tyrosines have become an interesting target for facile site-selective modification of proteins due to their moderate abundancy at the surface of proteins and hence limited solvent accessibility. Extensive study of tyrosine modification by the group led by Francis in the early 2000s led to the development of valuable chemoselective strategies and reagents—e.g. diazonium reagents, three-component Mannich-type reactions, palladium-catalysed *O*-alkylation—which were shown to lead to site-selective modifications of different proteins, from bacteriophage MS2 to chymotrypsinogen A [73,251,252].

**Figure 17.**
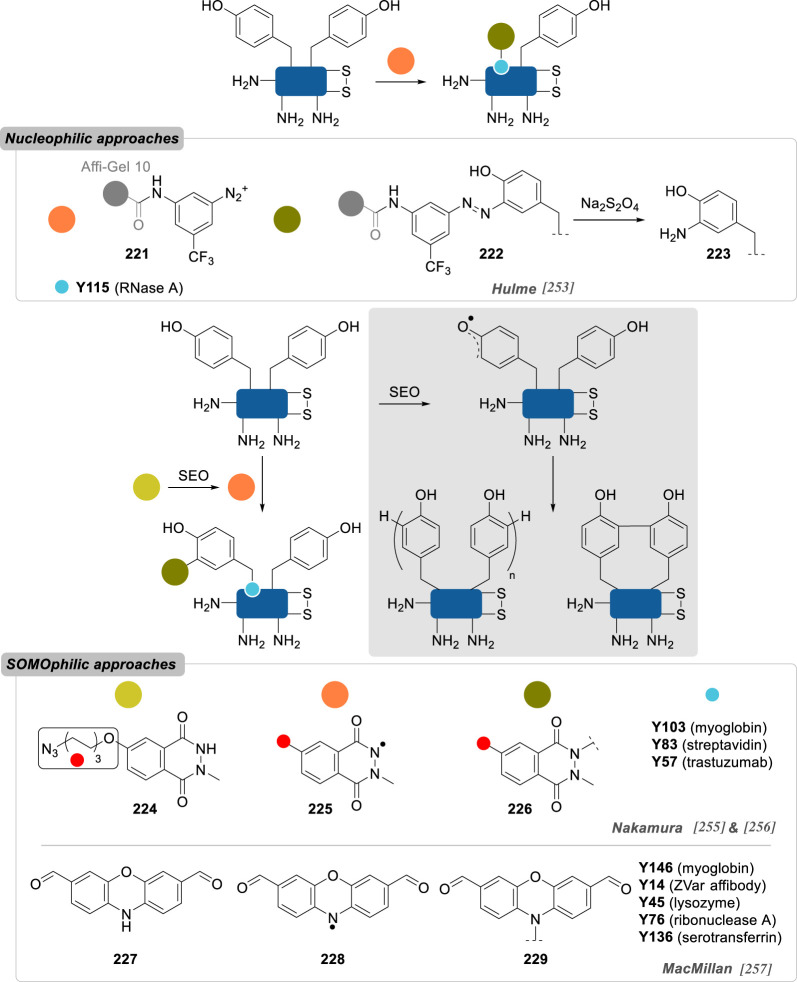
Overview of site-selective chemical conjugation of native proteins on tyrosine (SEO, single-electron oxidation).

More recently, Hulme and co-workers [253] developed an original catch-and-release strategy for the site-selective tagging of proteins (figure 17). Using agarose beads derivatized with diazonium groups **221**, they managed to trap proteins via electrophilic aromatic substitution of solvent-accessible tyrosine residues. Interestingly, they demonstrated that site-selectivity was possible, at least for RNase A, with only one (Y115) out of the three most exposed tyrosines being conjugated, which could be possibly explained by minimized electrostatic repulsion between the resin and the region around this residue. While the yield of protein capture was rather low—33% at best—addition of sodium dithionate led to the cleavage of the azobenzene group of **222** and the release of the modified protein, bearing an *o-*aminophenol group **223** that could be selectively oxidized and derivatized following Francis protocol (*vide supra*).

Besides ‘two-electron chemistry’ approaches relying on nucleophile/electrophile duets, another prominent strategy for tyrosine modification has taken advantage of the SOMOphilic character of the phenol ring—its propensity to react selectively with radical species. While single-electron oxidation (SEO) of tyrosines' phenol ring to *C*-centred radical species has been used to produce cross-linked proteins and protein oligomers (see box, figure 17) [254], a similar approach has been employed by different groups to generate reactive *N*-centred radical reagents—either enzymatically, using a combination of horseradish peroxidase (HRP) and hydrogen peroxide, or via electrochemical and photochemical techniques—[255] in order to label selectively only surface-exposed, hydrogen-bonded residues. Nakamura and co-workers [256] employed *N*-methylated luminol radical derivatives **224** and observed excellent chemo- and site-selectivity on different proteins, including antibodies such as trastuzumab in which Y57 was identified as the unique conjugation site [255]. However, site-selectivity was lost when multiple surface-exposed residues were present, as in the case of rituximab where four tyrosines were found to be responsible for the antibody's conjugation (Y32, Y52, Y101 and Y102). Very recently, the MacMillan group detailed the photoredox-catalysed coupling between tyrosines and phenoxazines **227**. When exposed to blue light (34 W, 440 nm) in the presence of lumiflavin as the photocatalyst, the phenoxazine ring was converted to the electrophilic *N*-centred radical **228** that coupled selectively with the electron-rich phenol groups of tyrosines. In addition, the presence of two formyl groups on the phenoxazine ring allowed for further functionalization of the conjugates by classical hydrazine ligation [257]. Most notably, this strategy led to exquisite site-selectivity on nine different native proteins with molecular weights ranging from 5.8 to 77.0 kDa. The observed selectivity was rationalized by the preferential reactivity of phenol side-chains engaged in hydrogen bonding with their local microenvironment and exposed to the surface of proteins. Conversions were higher than 70% on average and site-selectivity levels essentially over 90%, with a single labelled tyrosine identified in five cases—in the most prominent example, Y136 was the sole conjugation site identified on serotransferrin (77.0 kDa), despite the presence of 25 other tyrosines. Curiously, while following a very similar approach based on the same rationale (i.e. surface-exposed tyrosines react best), Nakamura and MacMillan identified two different conjugation sites on horse myoglobin (which only possesses two tyrosine residues): Y146 for MacMillan, Y103 for Nakamura. The absence of reactivity of Y146 in the latter case was suggested to be due to its shielding from **225** by the heme-coordinated H93 residue—we could imagine that the same heme unit could direct the preferential approach of phenoxazine radical **228** towards Y146 via electrostatic interactions and explain MacMillan's study results.

Turning to another amino acid residue equipped with an electron-rich side-chain, tryptophan has also been envisioned as an attractive target for site-selective modification. Being the least abundant proteinogenic amino acid in proteins—to the exception of the exotic pyrrolysine and selenocysteine—chemoselectivity and site-selectivity could, therefore, coincide in the case where only one tryptophan residue is present in the POI. Over the years, different research groups have tried to modify this amino acid selectively, albeit with limited success on proteins due to the harsh conditions being often required. Two main families of reagents have been investigated for this purpose: metallocarbenes and radical species.

**Figure 18.**
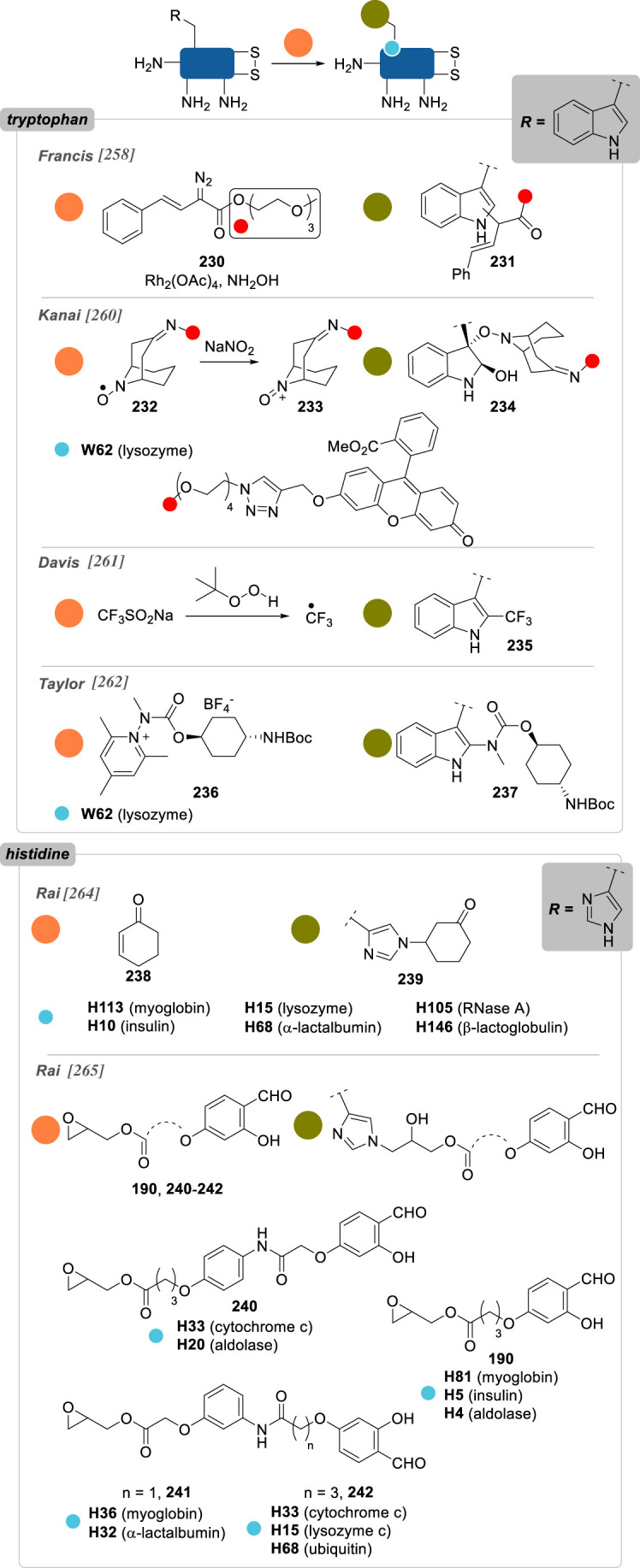
Overview of site-selective chemical conjugation of native proteins on tryptophan and histidine.

In 2004, Antos & Francis [258] proposed using metallocarbenes—generated from rhodium(II) acetate and diazo precursor **230** in the presence of hydroxylamine as ligand for the intermediate complex—for the chemoselective modification of the indole rings of tryptophans—conjugation products **231** obtained as mixture of *N*- and *C*2-alkylated products—on two proteins: myoglobin (two W residues) and subtilisin Carlsberg (one W residue) (figure 18). Due to the residues' poor solvent accessibility in these proteins, conjugation had to be performed under exceedingly harsh conditions—pH 3.5 for myoglobin, 1.5 for subtilisin Carlsberg—in order to denature the proteins and expose the indole rings, leading quite predictably to an absence of site-selectivity in the case of myoglobin. Replacing hydroxylamine with *N*-(*tert*-butyl)hydroxylamine did not alter the chemoselectivity of the method but allowed us to conjugate peptides such as melittin under milder conditions (i.e. pH 6.0) [259]. However, thermally induced denaturation was still needed to conjugate effectively native proteins with unexposed tryptophan residues.

Single modification of tryptophan on various native proteins was achieved by Kanai and co-workers [260] under smoother conditions by mixing five equivalents of the 9-azabicyclo[3.3.1]-nonane *N*-oxyl (ABNO) derivative **232** with three equivalents of sodium nitrite, presumably generating reactive oxoammonium species **233**. This resulted in the sole formation of mono-labelled conjugates with conversions oscillating between 11% and 64% on five different proteins—lysozyme (64%), myoglobin (53%), concanavalin A (11%), BSA (25%) and *β*_2_-microglobulin (15%). Site-selectivity was demonstrated by X-ray diffraction analysis of a lysozyme conjugate, highlighting W62 as the only conjugated tryptophan residue over the six present in this protein, presumably because of a less hindered environment.

Radical trifluoromethylation of tryptophans using sodium trifluoromethanesulfinate (NaTFMS or Langlois reagent) and *tert*-butyl hydroperoxide (*t*BuOOH) was also described by Davis and co-workers [261]. Good chemoselectivity was observed, with tryptophan being identified as primary modification sites—other aromatics-containing amino acids were also found to be partially labelled in some cases. Logically, this translated to a good level of site-selectivity on pantothenate synthetase—possessing a single tryptophan residue—which was lost with more complex targets—i.e. horse-heart myoglobin (two tryptophans, W7 and W14; both labelled) and lysozyme (six tryptophans, W28, W62, W63, W108, W111 and W123; all labelled except W111).

Taylor and co-workers [262] proposed photochemical reactions as a valuable strategy for the modification of these residues due to tryptophan's propensity to participate in photoinduced electron transfer events. Employing diversely functionalized *N*-substituted pyridinium salts **236** and UV-B irradiation in the presence of glutathione, chemoselective C2-carbamylation of tryptophans' indole ring was observed via a mechanism that remains elusive. Fine tuning of the reaction conditions allowed excellent conversions to be obtained on few peptides and led also—like Kanai's ABNO strategy—to the selective coupling of W62 on lysozyme (up to 94% conversion) even though double modification could also be detected, accounting for up to 25% of conjugated species. This second labelling event could, however, be located on the same W residue, as evidenced during the optimization study conducted on the peptide octreotide.

After tyrosine and tryptophan, the last member of the electron-rich-aromatics family of α-amino acids is histidine and its imidazole ring. Chemoselective modification of this residue is challenging due to the presence of more nucleophilic groups on proteins that can outcompete the imidazole ring for substitutions and to the absence of a particular reactive feature of this aromatic ring that could be used to label it selectively—such as phenols’ facile generation of *C*-centred radicals, or indoles' high molar absorptivity and propensity to undergo photoionization, for example. Consequently, general, broadly applicable strategies for histidine modifications have regularly showed chemoselectivity issues [263], circumscribing site-selective reactions to only a few examples.

In an attempt to expand the portfolio of histidine-selective reagents, Joshi & Rai [264] screened several electrophiles on the model protein ubiquitin, which contains only a single histidine residue, and identified 2-cyclohexenone **238** as the best candidate (46% conversion; figure 18). The presence of a ketone in the final conjugate **239** allowed further functionalization of the protein with alkoxyamine probes via oxime formation, which also helped suppress the reversibility of the conjugation reaction under physiological conditions (PB, 0.1 M, pH 7.0, 25 °C). This strategy was applied to the site-selective conjugation of several native proteins, from insulin (H10, 52%) to myoglobin (H113, 25%) and His-tagged proteins in cell lysates.

Based on their previously reported linchpin-directed modification (figures 11 and 14), Rai and co-workers [265] developed a variation adapted to the modification of histidines (figure 18). In this latter case, reagents with two reactive handles separated by linkers of different sizes and structures were designed (**240**–**242**). On one extremity, an aromatic aldehyde will form Schiff bases with lysines (fast, reversible); this will help to direct the approach of a monosubstituted epoxide—located on the other extremity—towards a proximal histidine to form *N*-alkylated adducts (slow, irreversible). Site-selective, monolabelling of various proteins was demonstrated, albeit on different histidines and in lower conversion compared with their cyclohexenone approach (*vide supra*) for the same targets. This degree of selectivity seems to depend on both the linker structure (no *N*-terminal labelling was identified with **190**, as opposed to what had been observed on RNase A and aprotinin; figure 14) and the protein size: while the Fab fragment of trastuzumab could be site-selectively conjugated (H189; light chain) with **190**, this site-selectivity eroded when the full antibody was used instead. Interestingly, single labelling of a unique protein (cytochrome C) could be achieved when reacted in a mixture with six other proteins.

Similar to tryptophan, methionine residues are also both scarce—the second least abundant amino acids in proteins—and poorly solvent accessible, tending to be buried deep into proteins' tertiary structures. Killing two birds with one stone, developing a methionine-selective conjugation strategy would thus likely lead to a site-selective approach. While limited, a few methods can be found in the early literature, essentially relying on *S*-alkylation at low pH, methionines' thioether groups being the sole side-chain residue not protonated under harsh acidic conditions. While such stringent conditions helped compensate for the low intrinsic nucleophilicity of the thioether group, the potential instability of the resulting sulfonium coupled with the absence of biocompatible conditions explained why this approach had been disregarded. Offering a new look at methionine conjugation, two seminal papers from the late 2010s reported smooth and chemoselective strategies for the labelling of this residue.

**Figure 19.**
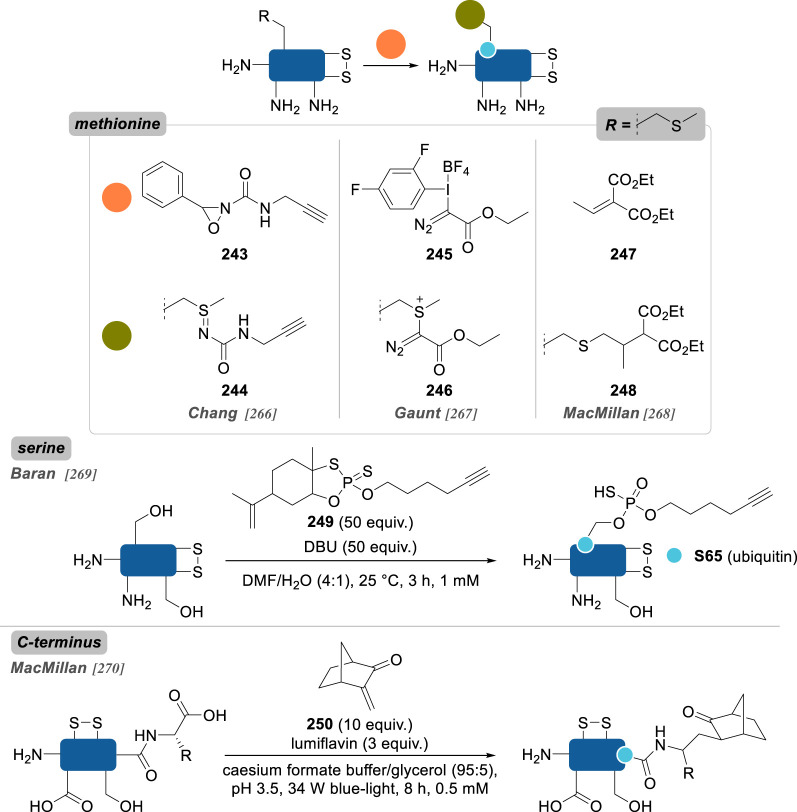
Site-selective strategies for the chemical conjugation of methionine, serine and *N*-terminal residues of native proteins (R, side-chain of proteinogenic α-amino acids).

In 2017, Toste, Chang and co-workers [266] detailed the use of oxaziridine reagents such as **243** for oxidizing thioether to sulfimides **244** within minutes at room temperature (PBS, pH 7.4; figure 19). Only surface accessible methionines were found to react, which resulted in the nearly quantitative conversion of all nine native methionine residues on native calmodulin to their sulfimide derivatives. The strategy proved to be highly versatile and was used to produce ADC—employing artificial trastuzumab though, due to the lack of solvent-accessible methionine in the wild-type antibody—and to identify proteins with reactive methionines in cell lysates in a chemoproteomic approach. One year later, in 2018, the group led by Gaunt reported an alternative strategy, this time employing hypervalent iodine(III) reagents such as *λ*^3^-iodane **245** [267]. The reaction proceeded in the presence of TEMPO—to minimize the formation of oxidative by-products—and thiourea—whose role was not fully understood—under acidic conditions, and led to the formation of sulfoniums **246** equipped with a diazo motif that could be further functionalized by photocatalysis. Here as well, the method proved to favour solvent-accessible methionines, and while excellent levels of monofunctionalization could be observed on various proteins, identification of the modified methionines was not discussed.

More recently, the MacMillan group offered a third original and effective strategy for methionine conjugation, based on a photoredox catalytic protocol similar to that of tyrosine conjugation with phenoxazines derivatives discussed previously (*vide supra*) [268]. In the presence of flavin as photocatalyst, methionines’ thioether functions could be converted to α-thio radicals under blue-light irradiation, and trapped with various Michael acceptors, such as diethyl ethylidenemalonate **247**. Under smooth conditions, a handful of proteins were shown to be chemoselectively conjugated on methionine residues, with acceptable site-selectivity levels in cases where more than one methionine residue is present—up to approximately 70% for myoglobin (M55 versus M131, 5 : 2) and human growth hormone (M14 versus M125 and M170, 11 : 5).

Finally, it is worth mentioning some isolated examples of novel strategies aiming at the conjugation of previously unreported residues.

Baran and co-workers [269] paved the way toward serine-selective conjugation with phosphorus(V)-based electrophile **249**. In the presence of DBU, modification of serine could be detected, both as free amino acid and residue in peptides, in high yield and great selectivity. Despite lower conversion (less than or equal to 40%), transposition of this method to ubiquitin delivered a mono-labelled conjugate with S65 as the sole modification site. In the authors' own words, these reagents still need to be optimized to make them more reactive, more stable in aqueous conditions (reaction is done in 80% of DMF) and compatible with cysteine residues.

Finally, based on the difference in oxidation potentials between the side-chain carboxylates of aspartate and glutamate and the *C*-terminal α-amino carboxylate, Macmillan and co-workers [270] envisioned that the latter could be selectively modified via single-electron transfer. Employing lumiflavin as a water-compatible photocatalyst, the *C*-terminus of three different native proteins was efficiently modified with a variety of α,β-unsaturated carbonyls, including 3-methylene-2-norbornanone **250**, via photoredox chemistry under highly acidic conditions (pH 3.5). While conversions were rather limited, this strategy proved to be able to differentiate between the A- and B-chain *C*-termini of insulin, the former being selectively alkylated. This could be due to a variation in oxidation potentials between the two groups or to a directing effect from a lipophilic region nearby the A-chain *C*-terminus, adsorbing the photocatalyst.

## 4. Conclusion

The chemical conjugation of proteins has thus gone a long way since its first applications in the tanning industry more than 150 years ago thanks to the progressive understanding of proteins’ structure and composition which fostered the development of chemical reagents and techniques with improved selectivity. Essentially centred on the most nucleophilic and solvent-accessible lysine and cysteine residues in the late 1990s, chemoselective strategies have benefitted from the marked interest of the community of synthetic chemists, which led to the development of various techniques for the precise labelling of functional groups that were previously inaccessible—e.g. methionines' thioethers, tryptophans' indoles, serines’ primary alcohols, *N*-ter and *C*-ter residues. In parallel, synthetic chemistry *and* synthetic biology both powered heavily the development of site-selective protein conjugation with the expansion of UAA mutagenesis and the rise of bioorthogonal reactions, resulting in an exponential growth of reports and applications in the past decade. While not as effective yet, chemical strategies for the site-selective conjugation of native proteins have also been developed, some of which even showed promising levels of protein selectivity. The challenge in the case of native proteins lies in the understanding of the slight variations in reactivity of the various surface-exposed reactive entities, governed by the three-dimensional conformation of the protein and the microenvironment surrounding the residues and affecting their nucleophilicity, basicity and access. Working with these constraints, innovation in the site-selective conjugation of native proteins has been driven by two main approaches: the development of new reagents (e.g. bifunctional probes, phosphorus(V)-based electrophiles, hypervalent iodine(III) compounds) and that of novel techniques (e.g. photocatalysis, linchpin-directed modifications), with a clear overlap between these two areas in several cases. While predicting what the future holds in store for the field is nearly impossible, as it might depend heavily on the applications of all reported strategies to date, one can assume that the involvement of new techniques and new fields of research can lead to improved conjugation strategies. The 2010s have seen a renewed interested in electrochemistry from the community of synthetic chemists, which could one day permeate the field of bioconjugation. As another underexplored technique, mechanochemistry could offer interesting perspectives. Having transitioned from an exotic oddity to an industrially applicable method, one can imagine it could lead to new reactivity profiles by modifying local microenvironments at the surface of the protein. As shown by a recent work from the Bernardes group [199], computational chemistry can help design tailored reagents for site-selective applications. Coupled with X-ray crystallography to decipher proteins' three-dimensional structure, it could facilitate our understanding of the key factors influencing reactivity and lead to new synthetic strategies taking advantage of the spatial proximity between two or more reactive residues to narrow down the potential number of conjugation sites. While several strategies have already been reported, one can finally expect an increased participation from transition-metal catalysis in protein bioconjugation, even though the nitrogen- and oxygen-rich structure of these biomolecules—and the presence of several conjugated π-systems—can complicate this approach. Put together, this could foster spectacular breakthroughs, leading one day to the conjugation of presumably unreactive substrates—the family of alkyl-substituted amino acids—in the same manner as Csp^3^-H activation has reshaped the field of C–C bond formation. Paradoxically, the uncertainty about the future direction of the field is also the source of one of the only certainties to date: the field of protein bioconjugation still requires continuous development of new and novel chemical techniques and reagents—even for already well-studied residues such as lysines and cysteines—for the very reason that we never know what beneficial applications they might one day have.

## Data accessibility

No novel data have been generated to perform this work. All data presented in this work have already been published in the scientific literature and can be found in the references mentioned.
